# Unconventional acoustic wave propagation transitions induced by resonant scatterers in the high-density limit

**DOI:** 10.1038/s41598-024-63910-2

**Published:** 2024-06-27

**Authors:** Bernard R. Matis, Steven W. Liskey, Nicholas T. Gangemi, Aaron D. Edmunds, William B. Wilson, Brian H. Houston, Jeffrey W. Baldwin, Douglas M. Photiadis

**Affiliations:** 1 Naval Research Laboratory, Code 7130, Washington, DC 20375 USA; 2 Naval Research Laboratory, Code 7100, Washington, DC USA

**Keywords:** Gels and hydrogels, Phase transitions and critical phenomena

## Abstract

Experiments on ultrasound propagation through a gel doped with resonant encapsulated microbubbles provided evidence for a discontinuous transition between wave propagation regimes at a critical excitation frequency. Such behavior is unlike that observed for soft materials doped with non-resonant air or through liquid foams, and disagrees with a simple mixture model for the effective sound speed. Here, we study the discontinuous transition by measuring the transition as a function of encapsulated microbubble volume fraction. The results show the transition always occurs in the strong-scattering limit (*l*/*λ* < 1, *l* and *λ* are the mean free path and wavelength, respectively), that at the critical frequency the effective phase velocity changes discontinuously to a constant value with increasing microbubble volume fraction, and the measured critical frequency shows a power law dependence on microbubble volume fraction. The results cannot be explained by multiple scattering theory, viscous effects, mode decoupling, or a critical density of states. It is hypothesized the transition depends upon the microbubble on-resonance effective properties, and we discuss the results within the context of percolation theory. The results shed light on the discontinuous transition’s physics, and suggest soft materials can be engineered in this manner to achieve a broad range of physical properties with potential application in ultrasonic actuators and switches.

## Introduction

Doping soft materials with compliant, sub-wavelength impurities (including micron-scale impurities with nano-scale features) has been shown to be a controllable and scalable path forward for tailoring a material’s bulk effective ultrasonic properties^1–3^. Studies on doping soft media with non-resonant impurities have demonstrated the ability to continuously manipulate physical parameters like the acoustic index^1^, wave speed (longitudinal and shear), effective bulk modulus, and the frequency-dependent attenuation coefficient^2,3^. The ability to manipulate these properties is important for realizing novel acoustic materials, and for applications ranging from wearable sensors^4–6^ to micromachines^7–9^, medical devices^10,11^, and metamaterials^12–14^.

To study impurity-induced physics in soft materials, researchers often use hydrogels as a starting platform because gels are a model system affording long-term impurity suspension, a high acoustic impedance contrast with the impurities and a low acoustic impedance contrast with the surrounding environment (e.g., water), and negligible intrinsic loss^1,15–17^. Studies focusing on hydrogels doped with resonant impurities have shown large changes in the phase, group, and energy velocities, and have demonstrated conversion between coherent and incoherent energies within a strongly scattering regime (the mean free path is comparable to the wavelength), which are due to Mie scattering and the shifted Minnaert resonance for encapsulated microbubbles^16,17^. Equally important is these studies focus on dilute emulsions with impurity volume fractions of only a few percent, which is beneficial to the controlled fabrication of viscoelastic materials using pressure injection methods that rely on minimal changes in viscosity with the impurity addition^2^. Nevertheless, doping with resonant impurities generally leads to continuously tunable properties similar to doping with non-resonant impurities, and the resultant properties generally do not exhibit any discontinuous phase change behavior^2,3,15,16^.

However, recent measurements of ultrasound propagation through a suspending gel doped with gas-filled encapsulated microbubbles with a broad size (and therefore resonance frequency) distribution demonstrated a discontinuous change in sound speed (by a factor 2.5) at a critical excitation frequency^17^; moreover, broadband behavior (100’s kHz) was observed on either side of the critical frequency. Such discontinuous sound speed behavior disagrees with a simple mixture model (the so-called Wood’s model^18^ where $v={\left[\left(\Phi {\rho }_{fluid}+\left(1-\Phi \right){\rho }_{gas}\right)\left(\Phi {\beta }_{fluid}+\left(1-\Phi \right){\beta }_{gas}\right)\right]}^{-1/2}$ where *v* is the sound speed, Φ the fluid volume fraction, ρ the density, and *β* the compressibility), and also disagrees with multiple scattering models that predict a smoothly varying sound speed with changing dopant volume fraction^2,3^. Nevertheless, the discontinuous transition was not the primary focus of the previous work, and so the encapsulated microbubble volume fraction dependence of the observed discontinuous behavior, as well as the underlying physics, are still unknown. Gaining an understanding of such discontinuous behavior is important as hydrogels have received much attention owing to their ability, in part, to be used as building blocks in wearable sensors^4^, reversible switches^19^, and for their potential use in implantable microdevices with moving parts^7,8^. In this regard, the ability to remotely induce an abrupt transition within a gel’s properties could serve as a key component in such systems if the transition could be used as an actuator for moving parts or lead to switch-like behavior in material properties.

Here, we explore the wave propagation phenomenology of the discontinuous ultrasonic transition in encapsulated microbubble (EMB)-doped gel as a function of EMB equilibrium volume fraction ϕ over a large range of excitation frequency. It is shown that within the strong scattering limit (*l*/*λ* < 1, where *l* and *λ* are the mean free path and effective wavelength, respectively), the behavior of the frequency-dependent change in phase angle Δ*θ* switches abruptly from random-like to smooth at the critical excitation frequency, and the Δ*θ* versus frequency slope changes by a factor as high as 5.6 also at the critical frequency, which signals a clear change in system behavior. Across samples, and at the critical frequency, the longitudinal phase velocity changes discontinuously to $\overline{{v }_{L}}$ = 456 m/s with a standard deviation ${\sigma }_{{V}_{L}}=103$ m/s, which is intermediate between the gel and the EMB gas sound speeds, and we find *v*_L_ remains fixed with increasing ϕ for frequencies above the critical frequency. The observed ϕ-independent phase velocity is in stark contrast to the conventional ϕ dependence that occurs in our system for frequencies below the critical frequency, as well as in other systems with non-resonant air and hard sphere colloid suspensions, and indicates an abrupt change in the acoustic propagation mode (the mode changes from waves/excitations in the fluid being scattered by resonating EMBs to excitations of the EMBs being coupled to one another by the fluid). Moreover, in the low-ϕ limit, the critical frequency follows a power law ${f}_{C}^{*}\propto {\phi }^{b}$ with *b* = 2.715 ± 0.002, which we find is unlike the linear ϕ dependence for the frequency at which *l*/*λ* = 1 (which occurs at a lower frequency and is governed by the total amount of gas within the sample). The results cannot be explained by multiple scattering theory, viscous effects, mode decoupling, or a critical density of states, and we therefore hypothesize the discontinuous transition from fluid-like to gaseous-like behavior depends upon the microbubble on-resonance effective volume fraction, and we discuss the results within the context of percolation theory. Lastly, the timescale associated with the discontinuous behavior observed here is at least a factor 10^3^ smaller than the timescale associated with light-induced discontinuous volume transitions observed in gels (~ 5 ms)^20^, which is the current state-of-the-art in terms of discontinuous changes in such materials.

## Methods

**Figure 1:**
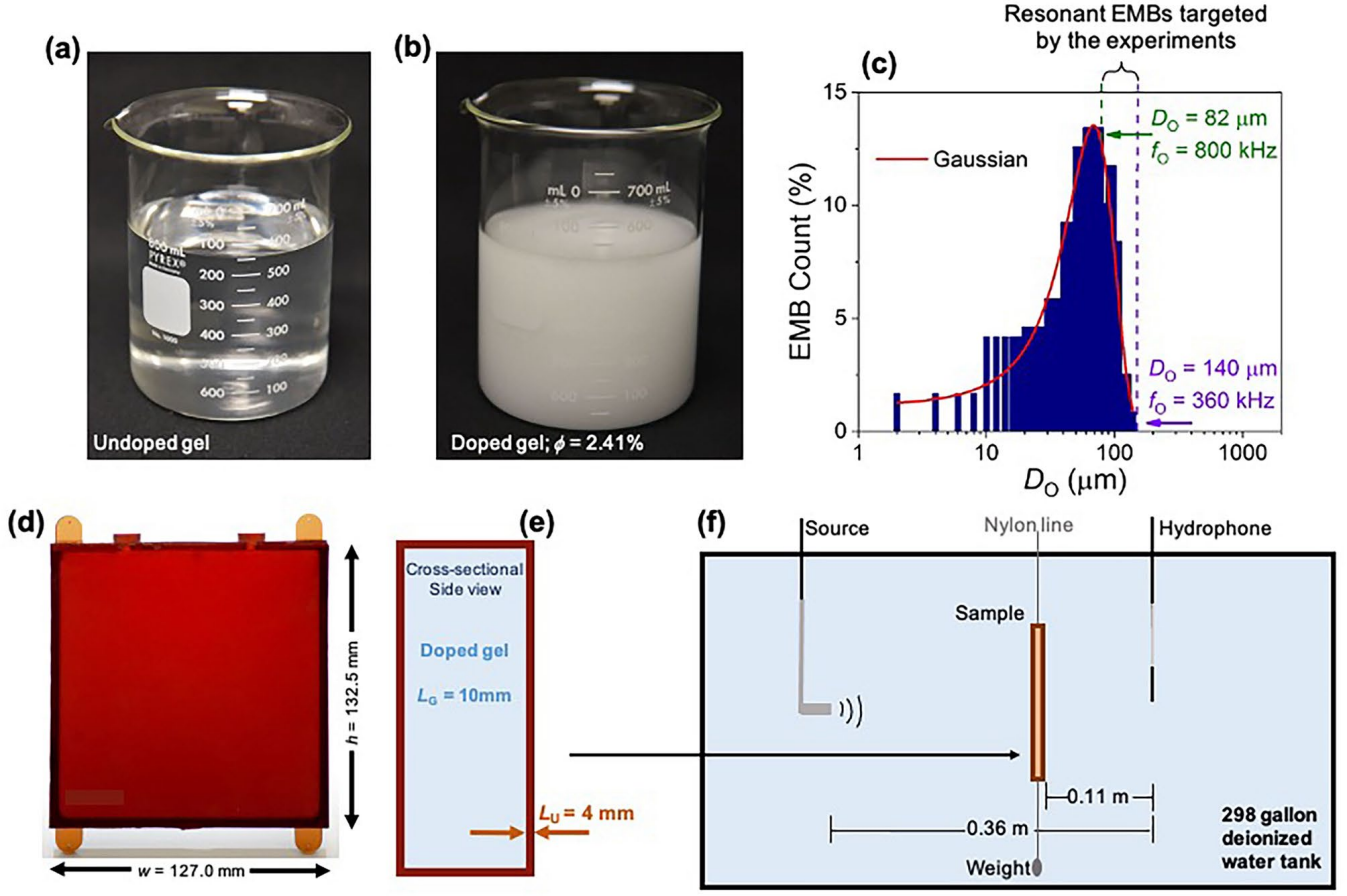
Encapsulated microbubble-doped gel and experimental setup. (**a**) Undoped gel image. (**b**) Image of the gel doped to an encapsulated microbubble (EMB) equilibrium volume fraction ϕ = 2.41% ± 0.05%, which was fabricated in the same manner as those gels for which data is presented. (**c**) EMB size distribution: EMB count (in percent) versus equilibrium diameter *D*_O_ determined with optical microscopy as presented in Ref. 17, and presented here with additional *D*_O_ and EMB resonance frequency *f*_O_ information as well as a Gaussian fit to the distribution (solid red line). The y-axis percentage reflects the number of EMBs at each *D*_O_ out of the total number of measured EMBs. Indicated is the *D*_O_ range, and the corresponding *f*_O_ range, targeted by the experiments. Note, the experimental frequency range is 50–800 kHz, which extends to frequencies well below the lowest *f*_O_ in the distribution. (**d**) The Uralite polymer shell into which the gel is poured for the in-water measurements. The center ports are sealed input/output through which the gel is injected. The top corner tabs are suspended from 0.6 mm diameter monofilament nylon line and the bottom corner tabs are weighted with two 63 g lead sinkers with a maximum dimension of 25.5 mm. (**e**) Sample cross-sectional side view showing the material thicknesses: *L*_G_ and *L*_U_ for the gel and Uralite layers, respectively. (**f**) Experimental setup showing the relative source, sample, and hydrophone positions for the in-water transmission measurements.

Ultrasound transmission and reflection measurements were performed on a suspending gel (an acoustic fluid) doped with resonant EMBs. The gel (Carbopol ETD 2050) was obtained from the Lubrizol corporation. The expanded EMBs (043 DET 80 d20) were obtained from Expancel. Each sample was made using EMBs drawn from the same batch, and optical images showing representative undoped and doped gels, as well as the optical contrast change that occurs with EMB doping, are presented in Fig. 1a,b. The measured EMB size distribution is well-fitted by a Gaussian, which is shown in Fig. 1c. Our experiments target the largest EMB diameters within the distribution, and there is a large overlap between the experiment’s frequency *f* range (50–800 kHz) and the EMB resonance frequency *f*_O_ range targeted by the experiments (360–800 kHz, see also Supplementary Fig. S1); for 50 kHz < *f* < 360 kHz, there are no resonating EMBs and so this frequency range serves as an additional reference to the behavior observed when the EMBs are resonating. Moreover, for *f* > 360 kHz the number of resonating EMBs at each frequency (i.e., the density of active oscillators ρ_active_) increases up to *f* = 800 kHz; thus, a key advantage of this system is the *in-situ* tunability of the disorder strength, which is controlled by varying the frequency over the broad EMB size (i.e., resonance frequency) distribution because only resonating EMBs contribute to the scattering. Moreover, the EMB size polydispersity index can be estimated from $\sigma /\overline{D }$, where $\sigma$ and $\overline{D }$ are the standard deviation and EMB average diameter obtained from the Fig. 1c Gaussian fit. Including all EMBs within the distribution yields a polydispersity index of ~ 37%. However, at a particular frequency only a fraction of the EMBs within the distribution are resonating, and for an EMB with an outer equilibrium diameter *D*_O_ = 90 μm, and based upon the full-width-at-half-maximum of the normalized scattering cross section versus frequency (see Supplementary Fig. S2) and the Fig. 1c Gaussian fit, we estimate the on-resonance polydispersity index to be ~ 8%.

**Figure 2:**
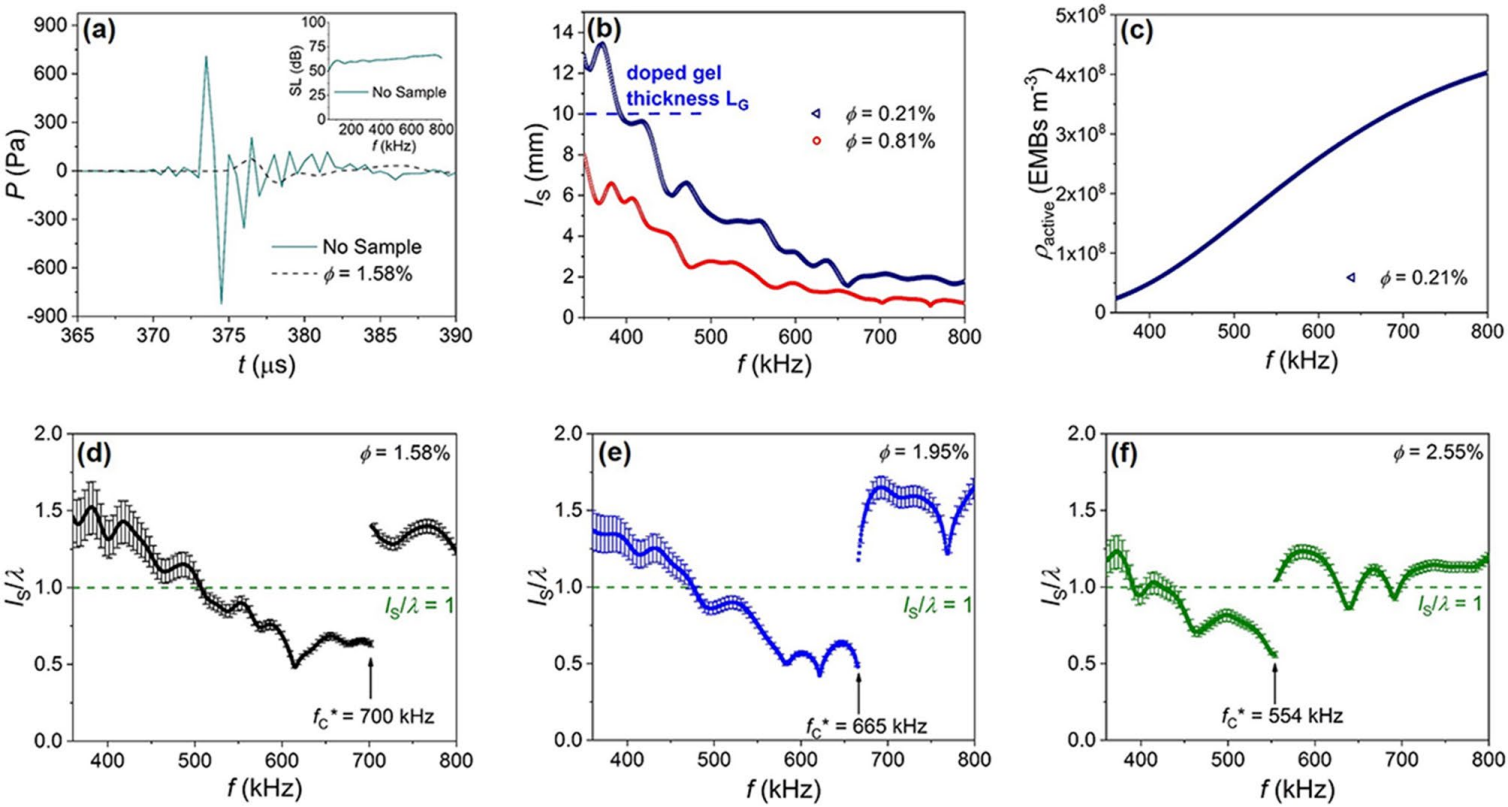
Wave propagation discontinuities in a strongly scattering medium. (**a**) Measured pressure *P* versus time *t* for the incident wavepacket (no sample, solid cyan line) and for transmission though the sample with an EMB equilibrium volume fraction ϕ = 1.58% (dashed black line). Inset: sound level SL frequency *f* spectrum for the incident wavepacket obtained during the water reference (no sample) measurement. (**b**) Measured scattering mean free path *l*_*S*_ versus *f* for the two doped samples with the lowest ϕ discussed within the main text. The dashed blue line is a reference for the doped gel thickness *L*_*G*_ = 10 mm. (**c**) Density of active oscillators ρ_active_ versus *f* for the sample with ϕ = 0.21%. (**d**)–(**f**) Frequency spectra for the ratio of *l*_S_ to the effective wavelength *λ* for three representative samples with different ϕ. For clarity, error bars (determined from uncertainties in the measured sound level and doped gel thickness) are shown for every 5th data point. The dashed green line is a reference to *l*_S_/*λ* = 1. In each figure part, *f*_C_* is the critical frequency at which a discontinuous transition is observed between wave propagation regimes (i.e., a transition between fluid-like and gaseous-like behavior). Also, in each figure part the horizontal axes are plotted over the EMB resonance frequency range targeted by the experiment.

The EMB resonance frequency within the gel is shifted from the Minnaert formula for a gas bubble in water due to the gel and EMB shell elastic properties; for example, an EMB with *D*_O_ = 90 μm (corresponding to *f*_O_ = 695 kHz in the gel) has *f*_O_ a factor of 10.7 higher than a bubble in water with the same diameter. On resonance the single EMB effective volume *V*_eff_ can be much larger than the equilibrium volume, and on resonance the EMB effective scattering cross section within the gel σe_ff_ is several orders of magnitude larger than the equilibrium geometrical cross section σ, and at each frequency scattering is dominated by resonating EMBs (for example, for *D*_O_ = 90 μm σe_ff_ = 237σ). In our system EMB damping is dominated by acoustic radiation damping within the frequency range of interest, which is unlike damping in such systems as liquid foams where there exists a nontrivial frequency-dependent absorption due to viscous and thermal effects^18,21^. See Supplementary Note 1 for information on EMB resonance frequency, scattering cross section, and damping. See Supplementary Note 2 for information on EMB density of states and the density of active oscillators (see also Fig. 2c).

The doped gel was encased in a soft, low-loss, undoped polymer shell made from Uralite 3140 (Fig. 1d,e); Uralite 3140 is acoustically transparent in water, has negligible attenuation and reflection in the frequency range of interest, and acts as a structural support for the gel during the in-water measurements. The Uralite 3140 parts A and B were acquired from Ellsworth Adhesives. The doped gel thickness *L*_G_ = 10 mm, and the Uralite shell into which the gel is poured has wall thickness *L*_U_ = 4 mm. Additional sample and fabrication details can be found in Ref. 17.

**Figure 3:**
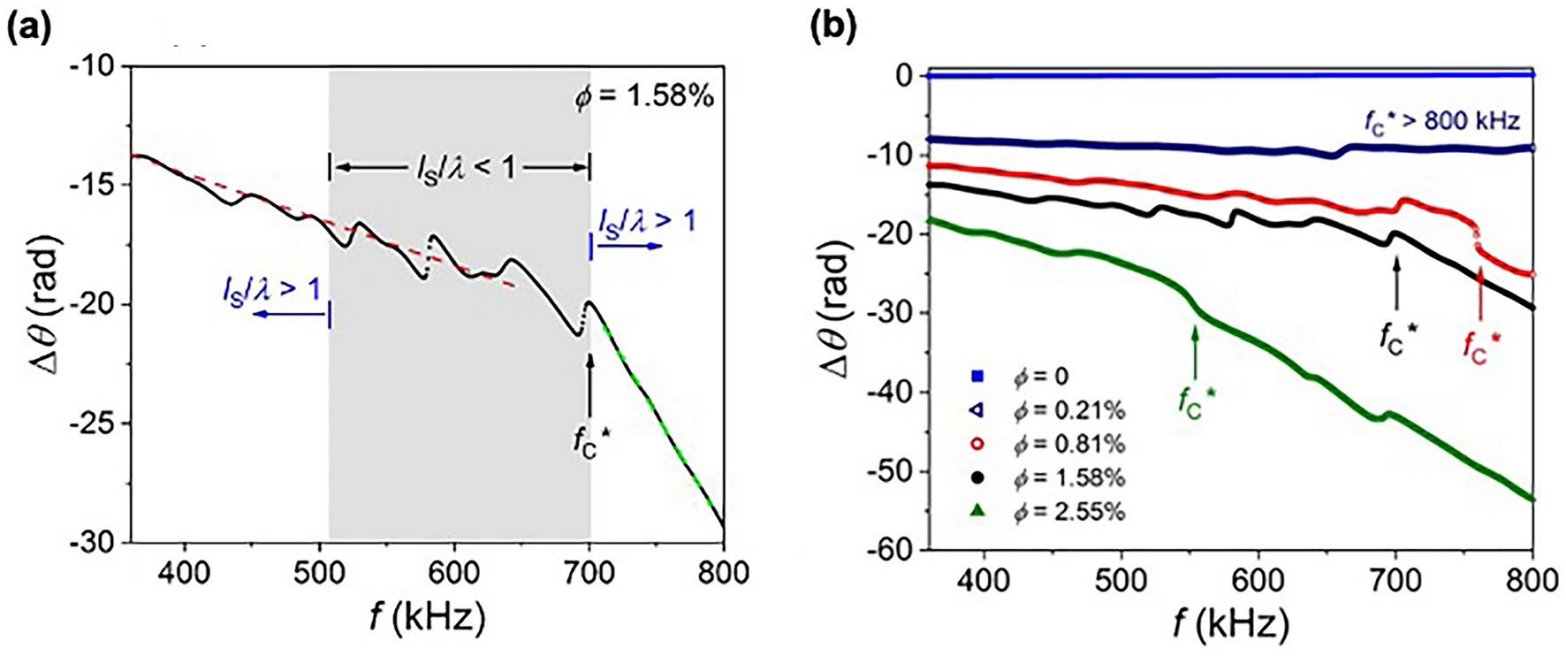
**(a)** Measured change in phase angle Δ*θ* versus frequency *f* for an EMB equilibrium volume fraction ϕ = 1.58%. The shaded gray region highlights the frequency range over which we measure *l*_S_/*λ* < 1 where *l*_S_ and *λ* are the scattering mean free path and effective wavelength, respectively (compare to Fig. 2d); outside the shaded gray region we measure *l*_S_/*λ* > 1. The dashed red and green lines are linear fits to the data from which we determine the slope of Δ*θ* versus *f* (and therefore phase velocity *v*_L_ versus *f*) on either side of the critical frequency *f*_C_*. (**b**) Δ*θ* versus *f* for varying EMB equilibrium volume fraction ϕ. The ϕ = 1.58% data set in (**b**) is the same solid black circle data set shown in (**a**). In each figure part, the horizontal axes are plotted over the EMB resonance frequency range targeted by the experiment. Also, in each figure part, *f*_C_* is the critical frequency at which the Δ*θ* slope markedly changes and the random-like nature of Δ*θ* versus *f*, which is most apparent when *l*_S_/*λ* < 1, becomes smooth.

The experiments were carried out in water, at normal incidence, within the temperature *T* = 297–299 K range, and utilized a 0.5 MHz piston-faced immersion transducer and a Reson TC 4035 hydrophone from Teledyne Marine. The in-water experimental setup used for the transmission measurements is shown in Fig. 1f. The experiments used a Krohn-Hite 5920 arbitrary waveform generator, and the waveform was filtered with an Ithaco 4302 dual 24 dB/octave filter and then amplified using an E&I 240L RF power amplifier prior to the waveform reaching the source transducer. The hydrophone signal was amplified using an Ithaco 1201 low-noise preamplifier before being digitized for data collection and processing (at a 2 MHz sampling frequency). For each data set, one thousand measurements were collected and averaged, and then a subtraction was used to eliminate any y-axis offset. A water reference was measured immediately prior to measuring each sample so both the reference and sample data sets were collected under identical conditions. We note this experimental setup has been used to measure the water sound speed temperature dependence, which is in agreement with standard published results for distilled water (see Supplementary Fig. S4), the properties of the undoped gel (see, for example, the Δ*θ* frequency spectrum for ϕ = 0 in Fig. 3b), and the properties of soft materials doped with non-resonating EMBs to comparable ϕ values^2^, and we have not observed discontinuous wave speed behavior in any of these prior reference measurements.

## Results

Figure 2a shows pressure *P* versus time *t* for the wavepacket recorded during the water reference (no sample) measurement, and for the coherent part of the transmitted wave for ϕ = 1.58% ± 0.05%; in this case, the maximum positive pressure decreases by a factor of 9.3 upon doping the gel with EMBs. Here, ϕ is determined through measurements of the undoped and doped gel densities using a graduated cylinder with a volume determined by a calibration measurement using water of known temperature and density, and by using an ideal mixture model for the doped gel density $\rho ={\phi }_{gel}{\rho }_{gel}+{\phi }_{EMB}{\rho }_{EMB}$; ϕ is also a measure of the total sample volume occupied by the EMBs in their equilibrium state and so each ϕ corresponds to a different sample. Also, shown in the Fig. 2a inset is the reference wavepacket’s measured sound level (SL) frequency spectrum, which shows the incident wavepacket exhibits a relatively flat response from 50 to 800 kHz, which aligns with the hydrophone’s usable frequency range (10–800 kHz). Figure 2b shows the scattering mean free path *l*_S_ frequency spectra for the two samples with the lowest ϕ (0.21% ± 0.04% and 0.81% ± 0.04%) where *l*_S_ is determined from the normalized intensity $I/{I}_{\text{O}}={e}^{-\left({L}_{\text{G}}/{l}_{\text{S}}\right)}$, *l*_S_ = (2*α*)^-1^ where *α* is the attenuation coefficient, and *I*_O_ is determined from a water reference measurement^22^. In our system, monopole scattering dominates, and so the scattering and transport mean free paths are equal. Figure 2b highlights how *l*_S_ decreases with increasing *f* above the minimum EMB *f*_O_ ~ 360 kHz to values considerably less than *L*_G_ = 10 mm (the smallest sample dimension), and this continuous decrease is due to an increasing ρ_active_ with increasing *f*. As an example, Fig. 2c shows ρ_active_ (determined through the measured EMB size distribution, EMB theory, and accounting for the finite σ(*f*) peak width) versus *f* for ϕ = 0.21%, and highlights how the number of scatterers contributing to the scattering increases with increasing frequency over the experimental frequency range.

Figure 2d–f show the measured *l*_S_/*λ* frequency spectra for varying ϕ: ϕ = 1.58%, 1.95% ± 0.05%, and 2.55% ± 0.05%; note, *l*_S_ and *λ *are determined from the transmitted coherent wave after time-windowing the data, and *v*_L_ and *λ* are determined from the measured Δ*θ* (see Supplementary Note 3 for information on Δ*θ*, *v*_L_, *λ*, and *l*_S_). Shown in Fig. 2d–f, *l*_S_/*λ* decreases with increasing *f* due to the increasing disorder strength with increasing frequency. Further, we observe in the strong scattering limit (*l*_S_/*λ* < 1) *l*_S_/*λ* exhibits a discontinuous rise to values above unity, and we label this critical excitation frequency *f*_C_*. We find *f*_C_* shifts to lower frequency with increasing ϕ: *f*_C_* = 700 kHz, 665 kHz, and 554 kHz for ϕ = 1.58%, 1.95%, and 2.55%, respectively. The discontinuous *l*_S_/*λ* rise at *f*_C_* occurs because *v*_L_ decreases at this frequency by a factor of about two, which agrees with prior results for the observed wave speed change at the critical frequency^17^. Additionally, at *f*_C_* = 665 kHz for ϕ = 1.95% we measured *α* = 933 Np/m, which is a factor 62 higher than *α* = 15 Np/m for the undoped sample (undoped gel in the Uralite-based shell), which exemplifies minimal attenuation due to the undoped materials in the absence of the EMBs.

Figure 3a shows the Δ*θ* frequency spectrum for ϕ = 1.58% where Δ*θ* is determined from the transfer function of the fast Fourier transforms for the sample and water reference data sets. As seen in Fig. 3a, random-like behavior in Δ*θ* versus *f* is observed with increasing frequency, and is most prominent within the frequency range where the condition *l*_S_/*λ* < 1 is satisfied; such random-like behavior indicates a strongly scattering material. However, the random-like Δ*θ* (*f*) behavior is not detected for *f* > *f*_C_* = 700 kHz. Also, *f*_C_* = 700 kHz is the frequency at which the Δ*θ* versus *f* slope changes by a factor of 4.9 (compare the slope of the dashed red and green lines in Fig. 3a), which indicates a change in material wave speed (for this factor of 4.9 change in slope, *v*_L_ is reduced from 874 m/s ± 4 m/s to 398 m/s ± 3 m/s). Thus, the absence of the random-like Δ*θ* (*f*) behavior for *f* > 700 kHz, along with the Δ*θ* (*f*) change in slope at *f*_C_*, indicates a clear change in the system’s effective properties with broadband behavior on each side of the critical frequency. We point out the discontinuous behavior is not the result of insufficient late-time data or signal processing. The Δ*θ* versus *f* data shown in Fig. 3a was determined from the wavepackets shown in Fig. 2a, and in our experiments data is collected for an additional 950 μs following the wavepacket’s arrival at the hydrophone (the water reference wavepacket, sculpted to have a flat SL across a specific frequency range, has a pulse width of ~ 20 μs and pulse length 1,492 m/s × 20 μs = 30 mm). Also, the data is time-windowed to avoid tank reflections, and we have confirmed the inclusion within the time-windowed data of an additional 182 μs after the wavepacket’s arrival (up to the first tank reflection) does not change the results (see Supplementary Fig. S5). Moreover, Fig. 3b shows Δ*θ* versus *f* for multiple samples (including for ϕ = 0 where no discontinuous behavior was observed), and these data sets highlight how *f*_C_* shifts to lower frequency with increasing ϕ, which suggests a critical parameter for the observed transition. For the Fig. 3b data, the Δ*θ* versus *f* slope changes at *f*_C_* by a factor of 5.6, 4.9, and 2.8 for ϕ = 0.81%, 1.58%, and 2.55%, respectively, between the different regimes.

**Figure 4:**
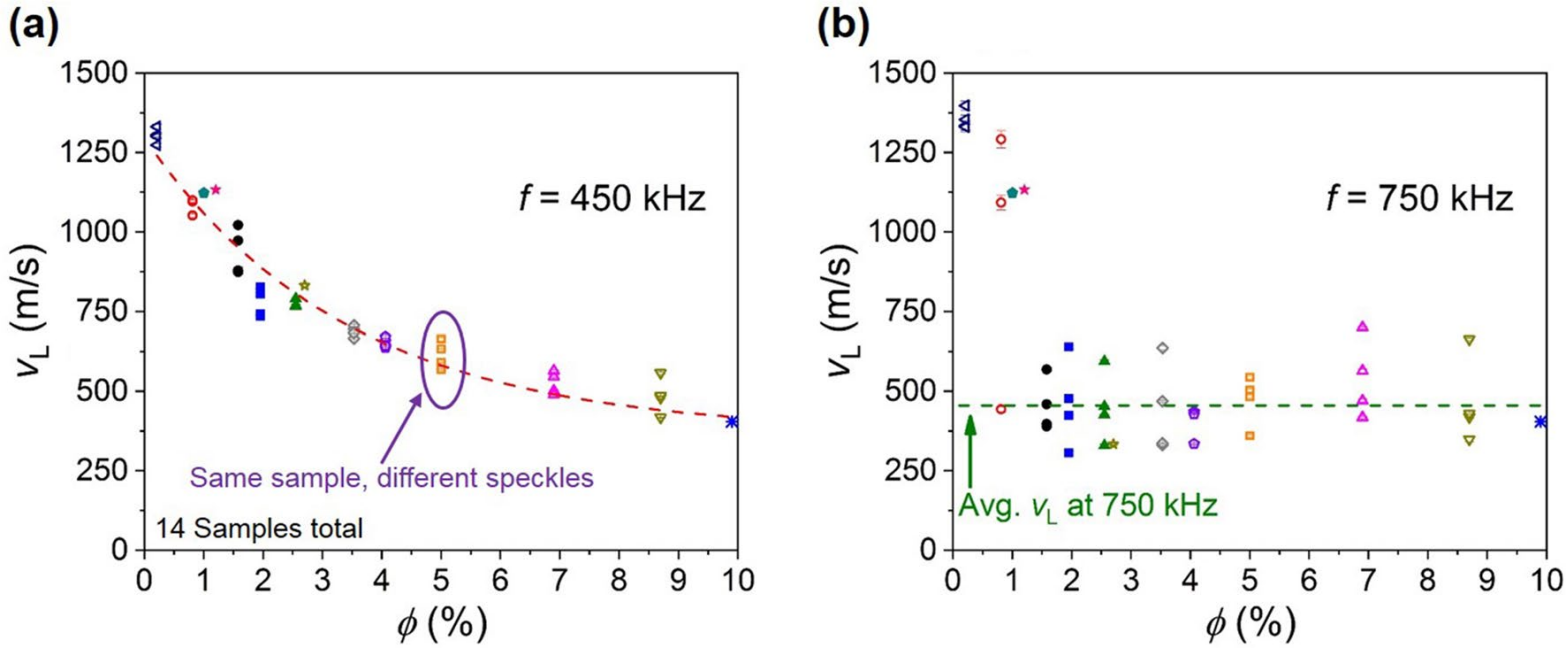
Phase velocity *v*_L_ versus EMB equilibrium volume fraction ϕ for two different frequencies: *f* = 450 kHz and 750 kHz in (**a**) and (**b**), respectively. At various ϕ we provide data for multiple independent speckle measurements. The dashed red line in (a) is a fit to ${v}_{L}\left(\phi \right)=a{e}^{-\phi /b}+{v}_{O}$ where *a* = 926 m/s, *b* = 3.39%, and *v*_O_ = 370 m/s. In (b), all *v*_L_ < 750 m/s correspond to the gaseous-like phase, and the dashed green line corresponds to *v*_L_ = 456 m/s, which is the average value of all data points in (b) for the gaseous-like phase where *v*_L_ < 750 m/s.

In Fig. 4a we plot *v*_L_ versus ϕ for *f* < *f*_C_* (specifically, at 450 kHz), and in Fig. 4b we plot *v*_L_ versus ϕ for *f* > *f*_C_* (specifically, at 750 kHz); note, in Fig. 4b there are a few exceptions at the lowest ϕ where we expect the discontinuous transition to occur at a frequency above our highest experimental frequency, however, all data points in Fig. 4b below 750 m/s correspond to the gaseous-like phase where *f* > *f*_C_*. Moreover, for each ϕ value in Fig. 4 we plot *v*_L_ values measured across speckles to provide a measure of the speckle-dependent spread in *v*_L_. For measuring within independent speckles, the sample was displaced within the plane perpendicular to the central axis from the source to the hydrophone, and we use displacements larger than the wavelength of sound in water at 500 kHz (*λ* ~ 3 mm). The sample was displaced so the source-hydrophone central axis traced a circle counterclockwise about the sample center. Furthermore, the hydrophone sensor’s aerial dimensions are smaller than the speckle coherence area (~ *λ*^2^), which ensures measurements within independent speckles. Figure 4a shows for *f* < *f*_C_* *v*_L_ can be fit with an exponential function for increasing ϕ. Contrarily, Fig. 4b shows for *f* > *f*_C_* *v*_L_ is ϕ independent (despite the increased spread in *v*_L_ across speckles) and has an average value $\overline{{v }_{L}}$ = 456 m/s with a standard deviation ${\sigma }_{{V}_{L}}=103$ m/s.

The most notable feature within the Fig. 4 data is that *v*_L_ is ϕ independent for *f* > *f*_C_*, which is unlike the behavior observed here for *f* < *f*_C_* where the strong dependence of *v*_L_ on ϕ is similar to that observed in soft materials where single and multiple scattering theories are applicable^2,3^. The ϕ-independent *v*_L_ is also unlike that observed in experiments measuring Mie scattering of monodisperse hard-sphere colloids^23^ where different excitations (resonant and interfacial) have phase velocities that vary with ϕ. We can rule out the *f*_C_* transition being governed by viscous effects because the viscous penetration depth *δ* is always less than *D*_O_ where $\delta ={\left(2\eta /\omega {\rho }_{l}\right)}^{1/2}$ and *η* and ρ_l_ are the gel viscosity and density, respectively, and *ω* = 2π*f*. That *δ* is always less than *D*_O_ across the frequency range of interest suggests *δ* is not an order parameter for the observed discontinuous behavior. Also, because *δ* is always less than *D*_O_ we can rule out the Biot theory for long-wavelength sound propagation in a porous medium that considers mode decoupling and sound propagation through the inhomogeneous fluid between the scatterers when *δ* becomes less than the pore size^24,25^. Further, Fig. 4b shows the measured *v*_L_ for *f* > *f*_C_* is not the increase one might expect to accompany wave propagation through the system’s inter-scatterer medium (*v*_L_ approaching that of the undoped gel), but instead is fixed at a value that is intermediate between that of the gel (1,498 m/s for the undoped sample) and the EMB gas (~ 200 m/s)^26^. We refer to the acoustic phase for *f* > *f*_C_* as “quasi-gaseous” because the resultant wave speed is very different from the pure fluid-like gel. See Supplementary Note 4 for information on the viscous penetration depth and Biot theory.

**Figure 5:**
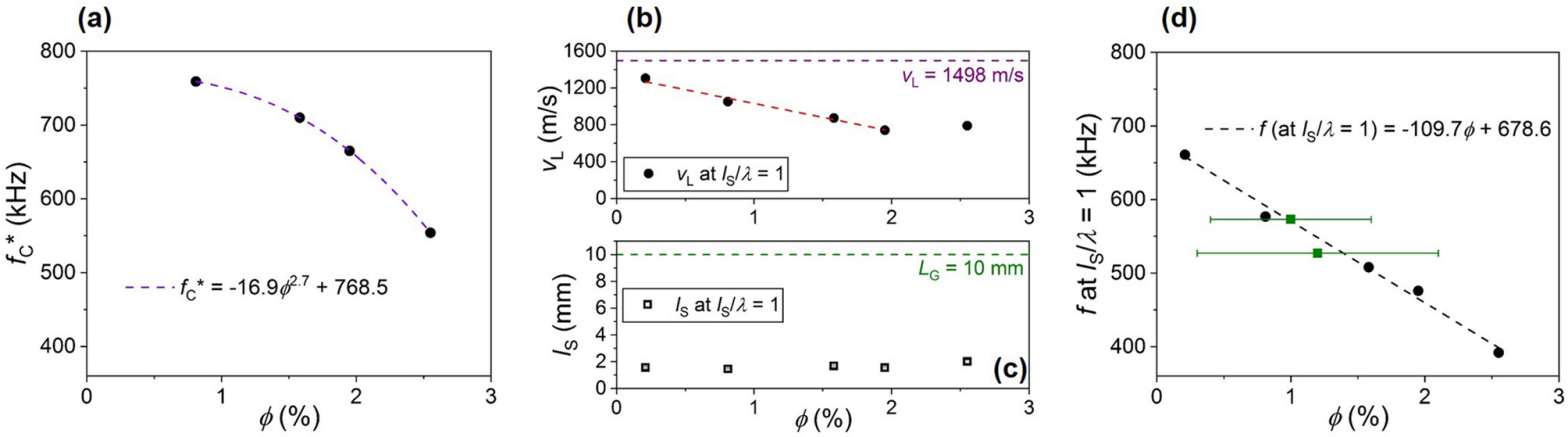
(**a**) Power law behavior of the critical excitation frequency *f*_C_* on EMB equilibrium volume fraction ϕ. The errors in *f*_C_* and ϕ are comparable to the solid black circle diameter. The dashed violet line is a fit to $y={y}_{O}+m{x}^{b}$ where *y*_*O*_, *m*, and *b* are fitting parameters. (**b**) Longitudinal phase velocity *v*_L_ at the lower frequency where *l*_S_/*λ* = 1 plotted versus ϕ (this frequency is determined by inspection of the *l*_S_/*λ* frequency spectra like those shown in Fig. 2d–f). The dashed red line is a linear fit to the data for ϕ < 2.0%. The dashed purple line is a reference for the undoped sample’s measured *v*_L_ = 1,498 m/s. (**c**) Scattering mean free path *l*_S_ at the lower frequency where *l*_S_/*λ* = 1 plotted versus ϕ. The dashed green line is a reference for the doped gel thickness *L*_G_ = 10 mm. (**d**) The frequency where *l*_S_/*λ* = 1 plotted versus ϕ. The solid green square data points correspond to samples studied in Ref. 17 (measured within the temperature *T* = 296–298 K range) while the solid black circle data points correspond to the samples studied in this work (measured within the *T* = 297–299 K range). The dashed black line is a linear fit with the fitting performed over the five data points with the lowest ϕ uncertainty (solid black circles).

Figure 5a shows *f*_C_* versus ϕ follows a power law in the low-ϕ limit: ${f}_{C}^{*}\propto {\phi }^{b}$ with *b* = 2.715 ± 0.002 determined from a least-squares fit. The observed power law behavior for *f*_C_* on ϕ is unlike the linear ϕ dependence measured for the frequency at which *l*_S_/*λ* = 1 (which occurs for *f* < *f*_C_*, and also shifts to lower frequency with increasing ϕ), which is shown in Fig. 5d. Moreover, Fig. 5b shows *v*_L_ at *l*_S_/*λ* = 1 also varies linearly with ϕ (in the low-ϕ limit where the *v*_L_(ϕ) exponential form shown in Fig. 4a can be approximated as linear), and Fig. 5c shows *l*_S_ at *l*_S_/*λ* = 1 is fairly constant across samples with an average value $\overline{{l }_{S}}=1.66$ mm (${\sigma }_{{l}_{S}}=0.23$ mm). Based upon *v*_L_ = *fλ* = *fl*_S_, the Fig. 5b–d data suggests the linear behavior for *f*(ϕ) at *l*_S_/*λ* = 1 shown in Fig. 5d is primarily the result of a linearly changing *v*_L_ with varying ϕ (a measure of the total sample volume occupied by the EMBs); this is qualitatively similar to the smoothly-varying changes in *v*_L_ that occur in systems even with non-resonant gas-filled impurities^2,3^. Additionally, the Fig. 5d, f versus ϕ slope (-109.7 kHz) is near (a factor 1.6 lower than) the slope of *v*_L_ vs. ϕ when divided by *l*_S_ (-180.9 kHz), which suggests a common origin for the linear behaviors shown in Fig. 5b,d; we also do not observe an abrupt change in *v*_L_ at *l*_S_/*λ* = 1. Contrarily, the *f*_C_* versus ϕ power law behavior suggests a different origin from a simple dependence on the overall EMB equilibrium volume fraction. Moreover, we can rule out the *f*_C_* transition being governed by a critical density of states *n*_*ω*_ (EMBs m^-3^ Hz^-1^) because *n*_*ω*_ varies by 66% at *f*_C_* between ϕ = 0.81% and ϕ = 2.55% (see Supplementary Fig. S7).

**Figure 6:**
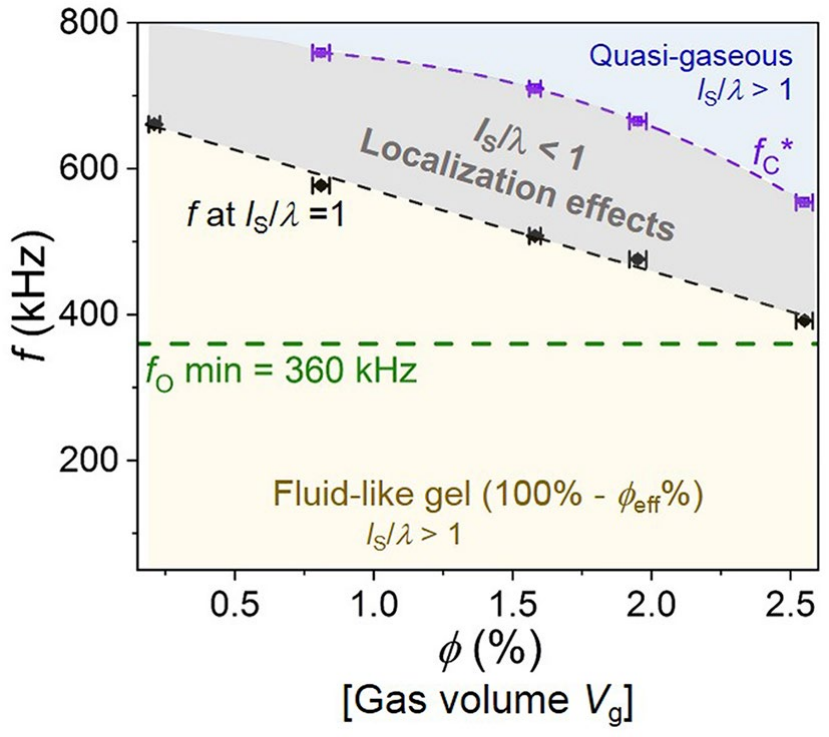
Ultrasonic phase diagram showing the binary transition between acoustic fluid-like and quasi-gaseous regimes. The diagram plots frequency *f* versus encapsulated microbubble (EMB) equilibrium volume fraction ϕ. The critical excitation frequency *f*_C_* at which the discontinuous transition is observed, plotted as open violet squares, are the same data points shown in Fig. 5a while the dashed violet line is the same fit shown in Fig. 5a. The solid black circles represent the lower frequency where *l*_S_/*λ* = 1, and are the same data points shown in Fig. 5d. The dashed black line is the same linear fit shown in Fig. 5d. The shaded gray region indicates the *f* and ϕ range over which we measure *l*_S_/*λ* < 1 where *l*_S_ and *λ* are the scattering mean free path and effective wavelength, respectively, as well as effects of wave localization. The shaded yellow region highlights the acoustic fluid (gel) phase, which can be expressed as 100%—ϕ_eff_% for increasing frequency where ϕ_eff_ is the EMB on-resonance effective volume fraction. The lightly shaded blue region indicates the quasi-gaseous phase (*f* > *f*_C_*). The dashed green line is a reference for the minimum EMB resonance frequency *f*_O_ ~ 360 kHz. For 360 kHz < *f* < *f*_C_ we observe diffusive wave propagation (not indicated).

Figure 4 shows the dependence of *v*_L_ on ϕ at two frequencies to exemplify the different sound speed behaviors on opposite sides of *f*_C_*. However, in our experiments we map the system’s acoustic phase diagram, which is realized by measuring the dependence of the frequencies *f*(at *l*_S_/*λ* = 1) and *f*_C_* on ϕ at constant temperature, external pressure, and sample volume. The Fig. 6 diagram shows *f*(at *l*_S_/*λ* = 1) and *f*_C_* versus ϕ, and since ϕ is the EMB equilibrium volume fraction it is also a measure of the total gaseous volume *V*_g_ if we ignore the approximately 100 nm-thick EMB shell; note, plotting the diagram with ϕ as the independent variable decouples the *x*-axis from the system’s frequency dependence as ϕ is simply a measure of the EMB fractional volume (which is frequency independent). Also shown on the Fig. 6 diagram are the same fittings shown in Fig. 5a,d (dashed violet and black lines, respectively). Thus, Fig. 6 shows a binary acoustic phase diagram, which consists of the fluid-like gel and quasi-gaseous phases, which are mediated by a range of *f* and ϕ over which we measure *l*_S_/*λ* < 1; thus, the region of the phase diagram over which *l*_S_/*λ* < 1 is satisfied can be thought of as a frequency-dependent transition region between the fluid-like and quasi-gaseous regimes. Moreover, the Fig. 4 data can be understood as slices of the diagram at constant frequency with the sound speed ϕ dependence indicated by the color shading. Lastly, the observed discontinuous behavior occurs along the dashed violet line connecting the Fig. 6*f*_C_* data points.

## Discussion

It is known that a first-order, reversible phase transition can be induced within a gel due to swelling and shrinking of the gel’s polymer network^20,27–31^. The discontinuous volume change can be induced by varying, for example, the gel’s temperature, solvent composition, or pH level, and can also be induced with exposure to visible light; in some cases, volume changes with swelling ratios near 10^3^ have been observed^27,28^. However, the volume expansion is slow and requires timescales on the order of hours to days for the system to reach equilibrium and for the transition to become discontinuous^27,29^. It is possible to reduce the timescale to several milliseconds with exposure to visible light^20^, however, such physics would be restricted to the light’s penetration depth within the gel’s host medium and would require a constant light source to maintain an equilibrium temperature. The Fig. 6 data shows the lowest *f*_C_* observed in our experiments for the discontinuous transition is 554 kHz, which corresponds to a period *T* = 1/*f* = 1.8 μs. Thus, the timescale associated with the discontinuous behavior observed here is at least a factor 10^3^ smaller than the timescale associated with light-induced discontinuous volume transitions observed in gels (~ 5 ms)^20^.

It is reasonable to associate *v*_L_ with the materials adiabatic bulk modulus *B* through *v*_L_ = (*B*/ρ_M_)^1/2^ where ρ_M_ is the constant equilibrium material density^32^, and so a discontinuity in *v*_L_ at *f*_C_* corresponds to a discontinuity in *B* and therefore a measured discontinuous change in material elastic properties. Further, the instantaneous potential energy density ${\mathcal{E}}_{p}$ is related to *B* through ${\mathcal{E}}_{p}=\frac{1}{2}\frac{{P}^{2}}{{\rho }_{M}{{v}_{L}}^{2}}\approx \frac{1}{2}B{s}^{2}$ where *s* is the condensation^32^ and so the system’s total internal energy density is also discontinuous at *f*_C_*. That *B* is intermediate between that of the fluid and EMB gas suggests the discontinuous changes in *B* and ${\mathcal{E}}_{p}$ are governed by an effective critical gas (i.e., EMB) volume fraction the frequency excitation of which couples the resonance behavior of the EBMs to one another by the surrounding fluid; in a sense, a dual system with interchanging roles of the two media.

In our system, the frequency-dependent EMB on-resonance effective volume fraction ${\phi }_{eff}\left(f\right)=\left(\pi /6\right)\left[{n}_{\omega }*\Delta \omega \right]{\left({D}_{eff}(f)\right)}^{3}$ can be significant where Δ*ω* and the EMB effective diameter *D*_eff_ are determined by EMB theory and the measured EMB size distribution. In Supplementary Note 5 we show for the range of *f* and ϕ over which the independent scattering approximation is valid (*f* < 600 kHz and the lowest ϕ = 0.21%) ϕ_eff_ can vary between 11.5 and 27.5%, which explains how such effects are observable in our samples despite the low EMB equilibrium volume fraction of a few percent. It is worth noting such high ϕ_eff_ are near the predicted three-dimensional percolation threshold volume fraction, which for most real mixtures falls within the 0.25–0.29 range^33^. We therefore hypothesize the observed discontinuous ultrasonic transition from fluid-like to gaseous-like behavior depends upon the microbubble on-resonance effective volume fraction, which reaches a percolation threshold when the EMB resonances become coupled by the surrounding fluid resulting in a discontinuous change in measured material properties. Nevertheless, future theoretical work is needed to explain the *f*_C_* versus ϕ power law behavior shown in Fig. 5a, and to determine whether such behavior is the result of coupled EMB resonances and a percolation transition.

Based upon the measured EMB size distribution (Fig. 1c), the smallest *D*_O_ is within the 1–10 μm range. An EMB with *D*_O_ = 1 μm has a predicted *f*_O_ = 660 MHz, and so for *f* > 660 MHz there are no resonating EMBs in our system. Thus, we anticipate the EMB size distribution to restrict the range of frequencies over which we will observe the quasi-gaseous phase since for *f* > 660 MHz the lack of resonating EMBs would imply a transition back to a fluid-like phase. That our experiment’s maximum frequency (800 kHz) is near the frequency associated with the maximum in the EMB size distribution suggests the system will return to a fluid-like phase within increasing *f* with a band of frequencies over which *l*_S_/*λ* < 1 is satisfied (mirroring the behavior observed between 50 and 800 kHz). Therefore, the broadband behavior observed here on either side of *f*_C_* could in principle be extended further in frequency.

As indicated on the Fig. 6 diagram, the range of *f* and ϕ for which *l*_S_/*λ* < 1 is also the range where we have observed effects of impurity-induced wave localization^17,34^, and in Supplementary Note 6 we provide a discussion and additional evidence for localization effects occurring within these samples for the range of *f* and ϕ specified. Supplementary Note 6 provides evidence for wave diffusion for *f* < *f*(at *l*_S_/*λ* = 1), a slowing of diffusion for *f*(at *l*_S_/*λ* = 1) < *f* < *f*_C_* consistent with the localization self-consistent theory, and a loss of the incoherent signal for *f* > *f*_C_* (indicating the absence of diffusive and localized states within this frequency band).

Lastly, it is known that the bubble response can be pressure dependent, and that linear and semi-linear theories are not valid in the regime of large amplitude bubble oscillations and in the presence of strong inter-bubble interactions^35–38^. We point out we have restricted our theoretical analysis to linear models for bubble dynamics due to the low driving pressures used in our experiments (Fig. 2a shows a maximum pressure less than 1 kPa). First, from the compressive force on a resonant bubble^39^ and the EMB shell shear modulus *G*_S_ ~ 1 GPa determined from prior works^2^ we estimate the ratio of the EMB outward displacement ζ to equilibrium radius *R*_O_ to be of order 10^–7^ for a 1 kPa driving pressure. We also estimate ζ/*R*_O_ in our case to be more than 20 times smaller than that for lipid-coated bubbles at the same driving pressure); lipid-coated bubbles have been used to demonstrate nonlinear effects within the 12.5–100 kPa driving pressure range where ζ/*R*_O_ can be of order 10^–4^ to 10^–3^, respectively (we obtain these ζ/*R*_O_ values for the lipid-coated bubbles using our same analysis and the shell shear modulus *G*_S_ = 45 MPa provided in Ref. 36]. Thus, we do not expect a nonlinear bubble response at the low driving pressure used in our experiments, which follows other experiments regarding sound transmission through gels doped with bubbles of comparable diameter and volume fraction^40^. Second, efforts have been made to develop nonlinear models that account for bubble–bubble interactions^36,38^, and these models predict sudden pressure induced changes in acoustic properties in the high-pressure limit that can shift to lower frequency with changing pressure and void fraction. However, the pressures considered in Ref.’s 36 and 38 are at least a factor of 10 higher than the maximum pressure used in our experiments, and the sudden changes have been predicted to occur primarily for pressures higher than 100 kPa. For these reasons, we restrict our analysis to linear models, and future pressure-dependent experiments can shed light on the contribution of nonlinear effects within the discontinuous acoustic transitions presented here.

## Conclusion

We have demonstrated a discontinuous ultrasonic transition between fluid-like and gaseous-like regimes as a function of encapsulated microbubble volume fraction. The results show the transition always occurs in the strong-scattering limit (*l*/*λ* < 1), the random-like behavior of the change in phase angle versus frequency becomes smooth at the transition’s critical frequency, that at the critical frequency the effective phase velocity changes discontinuously to a value that is constant with increasing microbubble volume fraction, and the measured critical frequency shows a nontrivial power law dependence on microbubble volume fraction. The results cannot be explained by multiple scattering theory, viscous effects, mode decoupling, or a critical density of states, and we hypothesize a percolation transition governed by the encapsulated microbubbles on-resonance effective volume fraction for the observed discontinuous behavior. The results shed light on the transition’s physics, suggest a broad range of tunable properties for soft materials with potential application in ultrasonic actuators and switches, and afford a system for studying resonant microbubble interactions at high effective volume fractions and possibly a percolation transition in three dimensions.

### Supplementary Information

Supplementary Information.

## Data Availability

The datasets used and/or analyzed during the current study available from the corresponding author on reasonable request.
