# Supplementary material for: Unconventional acoustic wave propagation transitions induced by resonant scatterers in the high-density limit
Source: Sci Rep. 2024 Jun 27;14:14872. doi: 10.1038/s41598-024-63910-2 (PMC11211437; doi:10.1038/s41598-024-63910-2)
Supplement: Supplementary file 1 — Supplementary Information. [file 41598_2024_63910_MOESM1_ESM.pdf]

# Supplementary Information

# Unconventional Acoustic Wave Propagation Transitions Induced by Resonant Scatterers in the High-Density Limit

Bernard R. Matis[1*], Steven W. Liskey[1], Nicholas T. Gangemi[1], Aaron D. Edmunds[1], William B. Wilson[1], Brian H. Houston[2], Jeffrey W. Baldwin[1], and Douglas M. Photiadis[1]

[1]Naval Research Laboratory, Code 7130, Washington, DC 20375, United States

[2]Naval Research Laboratory, Code 7100, Washington, DC 20375, United States

[*]Corresponding Author: bernard.matis@nrl.navy.mil (ORCID ID: 0000-0002-2681-6425)

**Supplementary Note 1: EMB resonance frequency, scattering cross section, and damping**

The encapsulated microbubble (EMB) linear harmonic oscillation is obtained from solutions of a Rayleigh-Plesset-like differential equation [S1], which considers damping due to acoustic radiation and the EMB shell viscosity. The EMB resonance frequency $f_O$ and angular frequency $\omega_O = 2\pi f_O$ prediction takes into account both the EMB shell and suspending gel densities ($\rho_S$ = 1,184 kg/m$^3$ for the EMB shell, and the gel density was determined separately for each sample prior to adding the dopants so that the EMB volume fraction $\phi$ could be obtained; for example, for $\phi$ = 0.81% the starting gel density was determined to be $\rho_l$ = 995.0 kg/m$^3$ ± 0.2 kg/m$^3$), the shell inner and outer radii (a shell thickness $\varepsilon$ = 100 nm is assumed based upon prior scanning electron microscopy measurements [S2]), the gel hydrostatic pressure (taken to be $P$ =101.325 kPa), the enclosed gas polytropic index ($\gamma \sim 1.1$), and the shell shear modulus ($G_S$ ~1.43 GPa) that was studied in prior work [S3]. The resonance angular frequency can be expressed in terms of the EMB equilibrium diameter $D_O$ as

$$\frac{1}{{\omega_O}^2} = \frac{{D_O}^2\left[\left({D_O}^3\rho_l\right)\big/_4 + \left({D_O}^2\varepsilon(\rho_S - 3\rho_l)\right)\big/_2 + D_O\varepsilon^2(3\rho_l - 2\rho_S) + 2\varepsilon^3(\rho_S - \rho_l)\right]}{{D_O}^3 3\gamma P + {D_O}^2 24 G_S\varepsilon - D_O 48 G_S\varepsilon^2 + 32 G_S\varepsilon^3} \qquad (S1)$$

Keeping terms to first order in $\varepsilon$ results in

$$\frac{1}{{\omega_O}^2} = \frac{{D_O}^2\left[\left({D_O}^3\rho_l\right)\big/_4 + \left({D_O}^2\varepsilon(\rho_S - 3\rho_l)\right)\big/_2\right]}{{D_O}^3 3\gamma P + {D_O}^2 24 G_S\varepsilon} \qquad (S2)$$

Keeping terms to first order in $\varepsilon$ is a good approximation to $\omega_O$: in the numerator the $\varepsilon$ term is over two orders of magnitude larger than the $\varepsilon^2$ term and over five orders of magnitude larger

than the $\varepsilon^3$ term, while in the denominator the $\varepsilon$ term is over three orders of magnitude larger than the $\varepsilon^2$ term and over six orders of magnitude larger than the $\varepsilon^3$ term.

In the limit that $\varepsilon$ goes to zero, we recover the Minnaert formula for a gas bubble [S4]: for $D_O$ = 90 μm, the Minnaert formula predicts $f_O$ = 64,850 Hz, which is far below the experiment's frequency range and does not explain the sound transmission properties of our samples between 50-800 kHz. However, to first order in $\varepsilon$ the resonance frequency can be written in terms of the EMB equilibrium radius $R_O$ as

$$f_O = \frac{1}{2\pi}\sqrt{\frac{3R_O\gamma P + 12G_S\varepsilon}{{R_O}^2[R_O\rho_l + \varepsilon(\rho_S - 3\rho_l)]}} \qquad (S3)$$

This expression yields $f_O$ = 695,055 Hz for $D_O$ = 90 μm, which is in agreement with the experimental results. Thus, to leading order in $\varepsilon$, the resonance frequency is shifted by the two new terms $12G_S\varepsilon$ and $\varepsilon(\rho_S\text{-}3\rho_l)$ resulting in a modified Minnaert formula due to the suspending gel and EMB shell properties.

For a specified EMB diameter the $\omega$-dependent scattering cross section $\sigma(\omega)$ is given by

$$\sigma(\omega) = 4\pi R_{10}^2\left(\frac{\rho_l^2}{\rho_S^2 a^2}\right)\left(\frac{\omega^4}{(\omega^2-\omega_O^2)^2+(2\beta\omega)^2}\right) \qquad (S4)$$

where $a = [1 + ((\rho_l - \rho_S)/\rho_S)\, R_{10}/R_{20}]$, $R_{10}$ and $R_{20}$ are the inner and outer EMB equilibrium radii, respectively, and $\beta$ is the total damping coefficient. Damping from acoustic radiation $\beta_{AR}$ is determined by

$$\beta_{AR} = \frac{\rho_l}{\rho_S a}\frac{\omega^2 R_{10}}{2v_O}\left[1+\frac{\rho_l^2}{\rho_S^2 a^2}\frac{\omega^2 R_{10}^2}{v_O^2}\right]^{-1} \qquad (S5)$$

where $v_O$ is take to be the measured (effective) phase velocity for each sample (i.e., for each value of $\phi$).

Supplementary Fig. 2 shows the predicted $\sigma(f)$ for a single, isolated EMB with $D_O$ = 90 μm (normalized to the equilibrium scattering cross section), which takes into account acoustic radiation damping. On resonance, the effective scattering cross section is several orders of magnitude larger than the equilibrium scattering cross section. Further, the inset to Supplementary Fig. 2 shows that acoustic radiation damping dominates the damping coefficient within the frequency range of interest, which is where the EMB resonance frequency range overlaps the experimental frequency range (primarily, 400-800 kHz). Damping from the EMB shell viscosity $\beta_{SV}$ is determined by

$$\beta_{SV} = \frac{2\mu_S(R_{20}^3 - R_{10}^3)}{a\rho_S R_{10}^2 R_{20}^3} \qquad (S6)$$

where $\mu_S$ ~ 10 Pa*s is the estimated PAN shell shear viscosity [S5]. The thermal damping coefficient for EMB oscillations [S6] is determined from

$$\beta_{TH} = \frac{9P_O\kappa(1+\Delta_P R_{10}/R_{20})^{-1}\left[G_+\left(\sqrt{Pe_g}\right) - 2/Pe_g\right]}{2\rho_S R_{10}^2(\kappa-1)\omega\left\{\left[3G_-\left(\sqrt{Pe_g}\right) + 1/(\kappa-1)\right]^2 + 9\left[G_+\left(\sqrt{Pe_g}\right) - 2/Pe_g\right]^2\right\}} \qquad (S7)$$

where $\Delta_P = (\rho_l - \rho_S)/\rho_S$, $Pe_g = 2\omega\rho_g c_g R_{10}^2/k_g$ is the gas Peclet number (for isopentane we use $\rho_g$ = 616 kg/m$^3$, $c_g$ = 1,660 J/kg*K, and $k_g$ = 0.11 W/m*K), and G± is given by

$$G_\pm\left(\sqrt{Pe_g}\right) = \frac{1}{\sqrt{Pe_g}}\left[\frac{\sinh\left(\sqrt{Pe_g}\right) \pm \sin\left(\sqrt{Pe_g}\right)}{\cosh\left(\sqrt{Pe_g}\right) - \cos\left(\sqrt{Pe_g}\right)}\right] \qquad (S8)$$

Lastly, in computations of $\sigma(\omega)$, $\beta_{AR}$, and the angular frequency dependent mean free path $l(\omega)$ it is useful to know the value of $D_O$ corresponding to each EMB $\omega_O$. We solve for $D_O$ as a function of $\omega_O$ by solving the following cubic equation, which follows from the modified Minnaert formula keeping only terms to leading order in $\varepsilon$:

$$D_O{}^3 + 2\varepsilon\left(\frac{\rho_S - 3\rho_l}{\rho_l}\right)D_O{}^2 - \left(\frac{12\gamma P}{\rho_l \omega_O{}^2}\right)D_O - \frac{96 G_S \varepsilon}{\rho_l \omega_O{}^2} = 0. \qquad (S9)$$

**Supplementary Note 2: The density of states and the density of active oscillators**

The number of EMBs on resonance at the resonance frequency $f_O$ can be expressed as

$$n_{EMB}(f_O) = n_{EMB_{tot}} * \left(distribution(f_O)\right) \qquad (S10)$$

where $n_{EMB_{tot}}$ is the total number of EMBs within a sample (i.e., for each value of EMB equilibrium volume fraction $\phi$). Through knowledge of the relationship between $D_O$ and $f_O$ (Supplementary Note 1) we can also write this expression as

$$n_{EMB}(D_O) = n_{EMB_{tot}} * \left(Gaussian\ distribution(D_O)\right) \qquad (S11)$$

where "Gaussian distribution($D_O$)" is the solid red line shown in the main text's Fig. 1c (thus, in computing the density of states $n_\omega$ we use the normalized Gaussian fit to the main text's Fig. 1c data). To compute $n_{EMB}(D_O)$ and therefore $n_{EMB}(f_O)$ it is necessary to determine $n_{EMB_{tot}}$ for each $\phi$ value. If all EMBs within a sample had the same diameter then the total EMB volume $V_{EMB_{tot}}$ is simply

$$V_{EMB_{tot}} = n_{EMB_{tot}} * V_{1EMB} = \phi V \qquad (S12)$$

where $V_{1EMB}$ is the volume of a single EMB and $V$ is the overall sample volume. In this case, since $V_{1EMB}$, $\phi$, and $V$ are known quantities this expression would give a simple way of determining $n_{EMB_{tot}}$: $n_{EMB_{tot}} = \phi V/V_{1EMB}$. However, not all EMBs in our samples have the same diameter, and so the measured distribution shown in the main text's Fig. 1c must be taken into account in determining $n_{EMB_{tot}}$. In a similar manner, we compute $n_{EMB_{tot}}$ through

$$n_{EMB_{tot}} = \frac{\phi V}{\int_{D_1}^{D_2} dD_O(Gaussian\ distribution(D_O))V_{1EMB}(D_O)} \qquad (S13)$$

where the upper and lower integral limits are taken to be $D_1 = 1$ μm and $D_2 = 140$ μm (corresponding to the lower and upper ranges of the measured EMB size distribution), and $V_{1EMB}(D_O) = (\pi D_O^3)/6$. With $n_{EMB_{tot}}$ known we can then compute $n_{EMB}(D_O)$ and therefore $n_{EMB}(f_O)$ using the above expressions and relationship between $D_O$ and $f_O$, which gives the number of EMBs at each $f_O$ for a given value of $\phi$.

To compute the density of states $n_\omega$ (i.e., the EMB density per Hz) we divide $n_{EMB}(f_O)$ by the factor $2\pi V(\mathrm{d}f_O)$ (i.e., $n_\omega = n_{EMB}(f_O)/\big(2\pi V(df_O)\big)$) where the quantity $\mathrm{d}f_O$ is computed by determining $\mathrm{d}f_O/\mathrm{d}R_O$ from Eq. S3 in Supplementary Note 1 which yields

$$\frac{df_O}{dR_O} = \frac{1}{4\pi}\left(\frac{3R_O\gamma P + 12G_S\varepsilon}{R_O^2[R_O\rho_l + \varepsilon(\rho_S - 3\rho_l)]}\right)^{-1/2}\Bigg[\frac{3\gamma P}{R_O^2[R_O\rho_l + \varepsilon(\rho_S - 3\rho_l)]} - \frac{(3R_O\gamma P + 12G_S\varepsilon)}{(R_O^2[R_O\rho_l + \varepsilon(\rho_S - 3\rho_l)])^2}\big[2R_O\big(R_O\rho_l + \varepsilon(\rho_S - 3\rho_l)\big) + R_O^2\rho_l\big]\Bigg] \qquad (S14)$$

Upon computing $df_O/dR_O$ we multiple by $dR_O$ in order to find $df_O$, where $dR_O$ = 10 μm is from the measured EMB count versus $D_O$ distribution bin size where the uncertainty in the EMB radius is ± 5 microns, which gives a total $dR_O$ = 10 μm. Supplementary Figure 3 shows the density of states $n_\omega$ versus resonance angular frequency $\omega_O$ squared for multiple EMB equilibrium volume fractions $\phi$ for which data is presented within the main text. In our experiments, $n_\omega$ versus $\omega_O^2$ is a one-sided, non-Gaussian distribution, which peaks at $f$ = 674 kHz. That the stiffness $K = m\omega_O^2$ where $m$ is the oscillator mass (which varies weakly across the distribution) implies $n_\omega$ versus $K$ is also non-Gaussian.

The density of active oscillators $\rho_{active}$ (EMBs m$^{-3}$) contributing to the scattering at each frequency $f$ can be found from the expression

$$\rho_{active}(f) = 2\beta_{AR}n_\omega = 2\pi(df_O)n_\omega \qquad (S15)$$

where $\beta_{AR}$ is the acoustic radiation damping factor given by Eq. S5 in Supplementary Note 1 and the quantity $2\beta_{AR}$ corresponds to the full-width at half-maximum of the scattering cross section (as exemplified by Supplementary Fig. 2). Figure 2c within the main text shows a representative plot of $\rho_{active}$ versus $f$ for $\phi$ = 0.21%, which highlights how the number of EMBs contributing to the scattering in our samples (i.e., the disorder strength) increases with increasing frequency.

**Supplementary Note 3: Measured change in phase angle, longitudinal phase velocity, effective wavelength, and scattering mean free path**

The longitudinal phase velocity $v_L$, determined from the coherent part of the transmitted wave, is found from the change in phase angle $\Delta\theta$ as a function of $f$ (see Fig. 3 of the main text for examples) where the phase spectrum is extracted from the transfer function of the fast Fourier transforms for the sample and water reference data sets. Values for $v_L$ are determined from $v_L = L/\left((L/v_W) - [(1/2\pi)(\Delta\theta/\Delta f)]\right)$ where $L$ is the material thickness and $v_W$ = 1,492 m/s the water sound speed. We determine $v_L$ = 1,480 m/s ± 10 m/s for the 4 mm-thick Uralite, $v_L$ = 1,498 m/s ± 5 m/s for the undoped gel sample (both the Uralite and Carbopol 2050 gel are closely impedance-matched to water), $v_L$ = 1,306 m/s ± 7 m/s ($f$ < 660 kHz) and $v_L$ = 1,398 m/s ± 14 m/s ($f$ > 660 kHz) for the sample with $\phi$ = 0.21%, $v_L$ = 1,052 m/s ± 5 m/s ($f$ < 702 kHz), $v_L$ = 670 m/s ± 7 m/s (702 kHz < $f$ < 759 kHz), and $v_L$ = 444 m/s ± 9 m/s ($f$ > 759 kHz) for the sample with $\phi$ = 0.81%, $v_L$ = 874 m/s ± 4 m/s ($f$ < 700 kHz) and $v_L$ = 398 m/s ± 3 m/s ($f$ > 700 kHz) for the sample with $\phi$ = 1.58%, $v_L$ = 742 m/s ± 4 m/s ($f$ < 665 kHz) and $v_L$ = 307 m/s ± 6 m/s ($f$ > 665 kHz) for the sample with $\phi$ = 1.95%, and $v_L$ = 791 m/s ± 5 m/s ($f$ < 554 kHz) and $v_L$ = 427 m/s ± 2 m/s ($f$ > 554kHz) for the sample with $\phi$ = 2.55%. These $v_L$ values are for individual speckles, and a measure of the spread in $v_L$ across speckles is shown in Fig. 4 of the main text. From measurements of $v_L$ we then compute $\lambda$ using the standard expression $\lambda = v_L/f$.

Additionally, we find $l_S$ decreases with increasing $f$ across the range of EMB resonance frequencies targeted by the experiments (specifically, 360-800 kHz) to values considerably less than the doped gel thickness $L_G$ = 10 mm. As an example, at the frequencies where $l_S/\lambda = 1$ for $\phi$

= 0.21% and 0.81% (661 kHz and 577 kHz for $\phi$ = 0.21%, and 0.81%, respectively) we measure $l_S$ = 1.57 mm and $l_S$ = 1.47 mm for $\phi$ = 0.21%, and 0.81%, respectively.

**Supplementary Note 4: Viscous penetration depth and the Biot theory for long-wavelength sound propagation**

For 360-800 kHz, the viscous penetration depth $\delta$ is always less than the EMB equilibrium diameter $D_O$ where $\delta = (2\eta/\omega\,\rho_l)^{1/2}$ and $\eta$ and $\rho_l$ are the gel viscosity and density, respectively, and $\omega = 2\pi f$ is the angular frequency. For a pH neutral Carbopol ETD 2050 gel (the suspending gel used in our experiments) the viscosity is expected to be $\eta \sim$ 10,000 mPa*s based upon the material's Technical Data Sheet, and we adopt this $\eta$ value in our determination of $\delta$. Supplementary Fig. 6 shows the frequency spectra for the EMB equilibrium diameter $D_O$ and the viscous penetration depth $\delta$. That $\delta$ is always less than $D_O$ across the full frequency range of interest suggests $\delta$ is not an order parameter for the transition into the quasi-gaseous phase as described within the main text.

Because $\delta$ is always less than $D_O$ we can rule out the Biot theory for long-wavelength sound propagation in a porous medium [S8, S9], which considers mode decoupling and sound propagation primarily through the inhomogeneous fluid when $\delta$ becomes less than the pore size: the Biot theory cannot explain an abrupt decrease in $v_L$ at $f_C$* because we do not observe $\delta$ become less than a critical parameter at this frequency.

**Supplementary Note 5: Effective volume fraction**

In determining the effective volume fraction, we first determine the range of $f$ and $\phi$ over which the independent scattering approximation (ISA) is valid. For weakly disordered/scattering systems the perturbative expression for the inverse mean free path $l^{-1}(\omega) \approx \sum_{\kappa} n_{\kappa}\sigma_{\kappa}(\omega)$ (with κ designating the scatterer type, $n_{\kappa}$ the scatterer density (scatterers per volume per frequency), and $\sigma_{\kappa}(\omega)$ the single scatterer cross section) leads to the association of attenuation resonances with single scatterer (Mie) resonances of type κ [S10]. For simplicity, we treat scatterers having different resonance frequencies as different scatterer types κ, assume the resulting scatterer types represent distinct scattering channels, and ignore any interference effects between the channels. Our EMB-doped system is different from Ref. S10 because the scatterer label κ is itself smooth and labels the scatterer frequency.

The angular frequency dependence of the inverse mean free path $l^{-1}(\omega)$ is derived by expressing the sum over the smooth index κ, which labels the EMB scatterer frequency, as the following integral

$$l^{-1}(\omega) \approx \sum_{\kappa} n_{\kappa}\sigma_{\kappa}(\omega) \approx \int_{0}^{\infty} d\omega_O n_{\omega O}\sigma_{\omega O}(\omega) \qquad (S16)$$

where $n_{\omega O}$ has units of EMBs/(m$^3$ Hz) and $\omega_O$ is the resonance angular frequency. Using the form of $\sigma(\omega)$ provided in Supplementary Note 1 (Eq. S4), the integral can be written as

$$l^{-1}(\omega) \approx \sigma_{\omega O}\omega^4 \int_{0}^{\infty} d\omega_O \frac{n_{\omega O}}{(\omega^2 - \omega_O^2)^2 + (2\beta\omega)^2} \qquad (S17)$$

where $\beta$ is the radiative damping factor and $\sigma_{\omega O} = 4\pi R_{10}^2\left(\rho_l^2/(\rho_S^2 a^2)\right)$ where $a = [1 + ((\rho_l - \rho_S)/\rho_S)\, R_{10}/R_{20}]$, $\rho_l$ and $\rho_S$ are the suspending gel and EMB shell densities, respectively, and $R_{10}$ and $R_{20}$ are the inner and outer EMB equilibrium radii, respectively. Rearranging the integral's denominator and factoring out an $\omega^2$ from each term in square brackets yields

$$l^{-1}(\omega) \approx \sigma_{\omega O} \int_0^\infty d\omega_O \frac{n_{\omega O}}{\left[\left(1 - \frac{\omega_O^2}{\omega^2}\right) + \frac{i2\beta}{\omega}\right]\left[\left(1 - \frac{\omega_O^2}{\omega^2}\right) - \frac{i2\beta}{\omega}\right]} \qquad (S18)$$

Making the substitution $y = \omega_O^2/\omega^2$, and for the resonance condition $\omega = \omega_O$, the integral becomes

$$l^{-1}(\omega) \approx \frac{\sigma_{\omega O}}{2}\omega \int_0^\infty dy \frac{n_{\omega O}}{\left[(1-y) + \frac{i2\beta}{\omega}\right]\left[(1-y) - \frac{i2\beta}{\omega}\right]} \qquad (S19)$$

By assuming negligible contribution to the integral for $-\infty < y < 0$ we can extend the limits of integration to $\pm\infty$ and integrate over the complex plane, which yields the expression

$$l^{-1}(\omega) \approx \pi n_\omega \sigma_{\omega O} \omega^2/(4\beta) \qquad (S20)$$

where the quantity $n_\omega$ has been found by evaluating $n_{O\omega}$ at $\omega$.

In computing $l^{-1}(\omega)$, $\beta$ is dominated by acoustic radiation damping with the damping coefficient $\beta_{AR}$ provided in Supplementary Note 1 (Eq. S5). Values for $\sigma_{\omega O}$ are computed knowing $R_{10}$ and $R_{20}$ as a function of frequency, which is determined by solving the cubic equation (Eq. S9) discussed in Supplementary Note 1 that considers all terms to first order in

EMB shell thickness $\varepsilon$. The density of states $n_\omega$ is determined by those procedures described in Supplementary Note 2.

The dashed pink line in Supplementary Fig. 8 is an ISA $l(f)/\lambda$ prediction where $l(f)$ is computed with Eq. (S20) and $\lambda$ is determined from the measured $v_L$. The ISA functional form only qualitatively agrees with the $\phi = 0.21\%$ data up to $f = 600$ kHz, and we find for $f > 600$ kHz and for higher $\phi$ the ISA no longer agrees qualitatively with the measured $l_S/\lambda$ functional form; note, the small decrease in the ISA prediction for $l(f)/\lambda$ at $f_C = 661$ kHz seen in Supplementary Fig. 8 is due to a measured increase in $v_L$ at this frequency, which effects both $\lambda$ and $\beta_{AR}$. The failure of the ISA to explain our data indicates significant multiple scattering occurs in our samples and that the ISA is largely inapplicable to our system (because the scatterers are resonant with effective size of order $\lambda$). Scattering in our samples is governed by on-resonance effective properties and samples with similar $\phi$ can have quite different effective volume fractions.

The frequency-dependent EMB on-resonance effective volume fraction $\phi_{\text{eff}}(f)$ is related to the density of states $n_\omega$ by

$$\phi_{eff}(f) = (\pi/6)[n_\omega * \Delta\omega]\big(D_{eff}(f)\big)^3 \qquad (S21)$$

where the quantity in square brackets represents the density of active (resonating) EMBs at frequency $f = \omega/2\pi$ (see Supplementary Note 2). We determine $D_{\text{eff}}$ from $\sigma_{\text{eff}}(f) = \pi R_{\text{eff}}^2$ knowing $D_O$ versus $\omega_O$ and computing $\sigma_{\text{eff}}(f)$ on resonance and accounting for acoustic radiation damping.

Note, at a given frequency, the effective scattering cross section is the result of scattering by the total number of active oscillators within the cross section peak width. Thus, to account

for a finite $\sigma_{\mathrm{eff}}(f)$ peak width in our computations of $D_{\mathrm{eff}}$ we determine the average effective scattering cross section $\sigma_{\mathrm{eff\_AVG}}(f)$ and then from $\sigma_{\mathrm{eff\_AVG}}(f)$ we compute an average effective diameter $D_{\mathrm{eff\_AVG}}(f)$. Integrating over the total scattering cross section in the effective medium (using the measured wave speed, as opposed to the undoped gel wave speed) gives

$$\sigma_{eff\_AVG}(f) = \frac{1}{\Delta\omega}\int_{\omega-\Delta\omega/2}^{\omega+\Delta\omega/2} \sigma_{eff}(\omega)\, d\omega = \frac{\pi}{16\omega_O^2 \beta_{AR}^2} \qquad (S22)$$

which yields $\sigma_{\mathrm{eff}}(f)/\sigma_{\mathrm{eff\_AVG}}(f) = 4/\pi$, and subsequently $D_{\mathrm{eff\_AVG}}(f) = [\sigma_{\mathrm{eff}}(f)]^{1/2}$. Values for $D_{\mathrm{eff\_AVG}}(f)$ are used to determine the effective volume fraction $\phi_{\mathrm{eff}}$.

The predictions shown in Supplementary Fig. 9 highlight that for a single EMB on resonance $D_{\mathrm{eff}}$ is significantly larger than $D_{\mathrm{O}}$ (between a factor of 15 and 19 greater depending upon the frequency). The predictions shown in Supplementary Fig. 10 highlight that in this case $D_{eff} \propto {}^{1}/_{f}$. Lastly, Supplementary Fig. 11 shows that within the range of $f$ over which the ISA is applicable, $\phi_{\mathrm{eff}}$ varies considerably (from 11.5% up to 27.5%), which explains how such effects are observable in our samples despite the low (a few percent) EMB equilibrium volume fractions. Also, the peak in $\phi_{\mathrm{eff}}$ versus $f$ near $f$ = 550 kHz is the result of an increasing EMB density of active oscillators $\rho_{\mathrm{active}}(f)$ and simultaneous decrease in $D_{\mathrm{eff}}(f)$ with increasing frequency.

**Supplementary Note 6: Reflection frequency spectra, Monopole scattering of longitudinal waves, incoherent wave data, additional SCT analysis, and absorption**

Supplementary Fig. 12 shows the measured reflected sound level (SL) versus $f$ for a 4 mm-thick undoped Uralite sample and for four doped samples with $\phi = 0.81\%$, $\phi = 1.58\%$, $\phi = 1.95\%$, and $\phi = 2.55\%$; note, the thickness of the Uralite sample was chosen to match the thickness of the walls of the Uralite shell into which the doped gel is poured for the in-water measurements. The SL minima observed in Supplementary Fig. 12 are explained in terms of thickness modes where the effective wavelength $\lambda$ becomes comparable to the material thickness, which results in a measured drop in the reflected SL at this condition. Despite the thickness modes, the overall maximum reflected SL for each sample is fairly constant across the entire experimental frequency range including those frequencies at which there are no resonating EMBs (50-360 kHz). We observe no evidence of resonant reflection across the entire frequency range of interest including at $f(l_S/\lambda = 1)$, $f_C$*, and for those frequencies where $l_S/\lambda < 1$ is satisfied. Also, internal reflectivity is weak and should not skew our late-time analysis because the ratio of the penetration depth $z_O$ to $L_G$ can be an order of magnitude less than unity: for example, at $f$ = 577 kHz for $\phi = 0.81\%$, $z_O = (2l_S[1 + R_r])/(3[1 - R_r])$ = 1.2 mm where $l_S$ = 1.47 mm and $R_r$ is the internal reflectivity [S11] estimated by averaging over the reflected sound level.

It is known from first principles that monopole scattering of longitudinal waves (expected in our system) does not create shear waves [S12], and so we do not expect slow shear waves to skew our late-time analysis of the incoherent energy. Even if near fields create higher-order multipole scattering, the coupling of longitudinal wave to shear wave is weak leading to an intensity reduction of order $10^{-4}$, and since wave energy must subsequently be converted back

into longitudinal waves to be observed an additional suppression of $10^{-4}$ is incurred; this is the result of the disparate wave speeds in our system (of order 1,000 m/s for longitudinal waves and of order 10 m/s for shear waves). Thus, such effects are expected to be negligible. Further, if shear waves were nevertheless generated by some unknown mechanism with appreciable amplitudes in our system (despite the fluid-like nature of our system), then we might expect them to be generated at all frequencies, even for frequencies where we observe diffusive effects (exponential decay of $I/I_O$ vs. $t$ where $I/I_O$ is the normalized intensity for the incoherent field and $t$ is the time) and for the quasi-gaseous phase (where the density of active oscillators is high) where we measure no incoherent energy above the experiment's noise floor. However, across samples, no late-time deviation from linearity is found when diffusive effects are measured (see Supplementary Fig. 14a for an example) or in the quasi-gaseous phase (750-800 kHz in Supplementary Fig. 14c-e), which supports no late-time component due to a shear wave.

To study late-time behavior of the transmitted incoherent energy we use a narrow impulse with width $\Delta t < 10$ μs. Supplementary Fig. 13 shows an example of measured pressure $P$ versus $t$ for $\phi = 2.55\%$ across speckles. We find the coherent field (266 μs < $t$ < 272 μs) is speckle independent. However, a strong incoherent field is observed for $t > 272$ μs, which varies across speckles and shows temporal fluctuations that vary on a time scale corresponding to the input wavepacket. Such fluctuations are the result of wave interference along multiple scattering paths [S11, S13, S14].

Supplementary Fig. 14a exemplifies how $I/I_O$ on a semi-logarithmic plot follows a linear time dependence for $f < f$(at $l_S/\lambda = 1$), which indicates diffusive wave propagation. Classically, at late times $I/I_O = e^{-t/\tau_D}$ where $\tau_D^{-1}$ is the lowest eigenvalue of the diffusion operator $-D\nabla^2$

[S15]. The Supplementary Fig. 14a data agrees with the diffusion model, and we obtain $\tau_D$ = 8.6 μs ± 0.5 μs, which yields $D^* = {L_G}^2/(\pi^2\tau_D)$ = 1.18 m$^2$/s ± 0.07 m$^2$/s ($L_G$ = 10 mm is the doped gel thickness) and a diffusion length $l_D = (D^*\tau_D)^{1/2}$ = 3.2 mm. Here, $l_D$ is over a factor of 3 smaller than $L_G$. Additionally, we have confirmed that changing the frequency range within the diffusive regime ($f < f$(at $l_S/\lambda = 1$)) leads only to linear $I/I_O$ versus $t$ behavior.

The Supplementary Fig. 14b,d&e solid symbol data sets show for $f$(at $l_S/\lambda = 1$) $< f < f_C$*, corresponding to the range of $f$ and $\phi$ where we measure $l_S/\lambda < 1$, we find deviations from diffusion at late times that are well-fitted by the phenomenological self-consistent theory (SCT) of localization [S16]: in a localized phase the average transmission coefficient $T(t) \sim e^{-\eta t}/t^{p+1}$ where $\eta = (D_B/\xi^2)\exp(-L_G/\xi)$, $D_B$ is the bare diffusion coefficient, and $\xi$ the localization length. The restriction $0.5 \leq p \leq 1.0$ yields $\xi$ = 2.41 mm, 2.35 mm, and 2.18 mm for $\phi$ = 1.58%, 1.95%, and 2.55%, respectively (more than a factor of 4 smaller than $L_G$). Also, in our fittings to the SCT, the bare diffusion coefficient $D_B$ serves as a free-fitting parameter, and we determine $D_B$ = 18.1 m$^2$/s, 10.1 m$^2$/s, and 23.4 m$^2$/s for $\phi$ = 1.58%, 1.95%, and 2.55%, respectively; these large $D_B$ values are inconsistent with our measured $D$*, but are consistent with prior studies on EMB-doped gel [S2] and sound localization in mesoglasses [S17].

Supplementary Fig. 14c,d&e show results for $f > f_C$*; specifically, for 750-800 kHz, which corresponds to frequencies where we measure a $v_L$ reduction, $l_S/\lambda > 1$, and the quasi-gaseous phase as described within the main text. Accounting for a 20 μs uncertainty for the 50 kHz digital filter applied to study the quasi-gaseous phase data, the data in Supplementary Fig. 14c,d&e shows no late-time evidence for diffusive or localized states for $f > f_C$* above the experiment's noise floor.

We do not expect absorption to skew our late-time analysis because the characteristic absorption time $\tau_a \geq 100$ μs is an order of magnitude larger than $\tau_D$ (which is found by multiplying $T(t)$ within the SCT by the factor $e^{-(t/\tau_a)}$). Further, taking $\tau_a \sim \tau_D = 10$ μs cannot account for the observed late-time deviations from linearity, and this is demonstrated by the dashed purple lines in Supplementary Fig. 15 for representative $\phi$ values. We conclude that absorption is unimportant in our measurements. Further, $f_O = 800$ kHz (the maximum frequency in our experiments) corresponds to a resonating EMB with $D_O = 82$ μm, which is near the maximum in the EMB size distribution shown in the main text's Fig. 1c. Yet, despite the continuous increase in the density of active oscillators with increasing frequency the loss of the incoherent signal for $f > f_C^*$ further supports the conclusion that absorption does not skew our late-time analysis since we would expect an increasing scatterer density might lead to higher absorption levels and persistent linear late-time behavior (which is not observed experimentally).

**Supplementary Figures:**

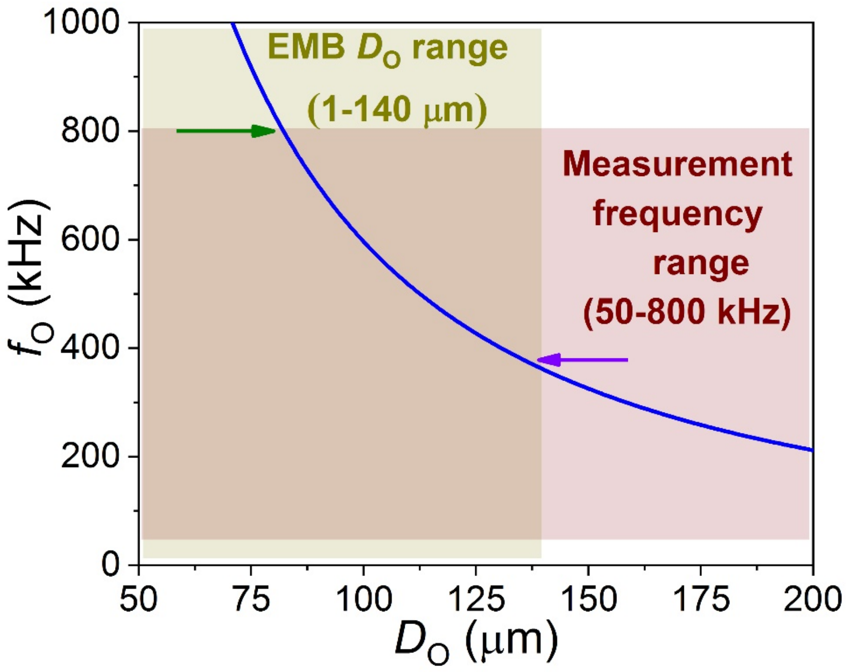


**Fig. S1** Predicted encapsulated microbubble (EMB) resonance frequency $f_O$ versus equilibrium diameter $D_O$ indicating the broad overlap (starting at ~360 kHz) between the experimental frequency range (50-800 kHz) and the $f_O$ range afforded by the distribution shown in Fig. 1c of the main text. The solid green and purple arrows indicate the same frequencies shown in the main text's Fig. 1c.

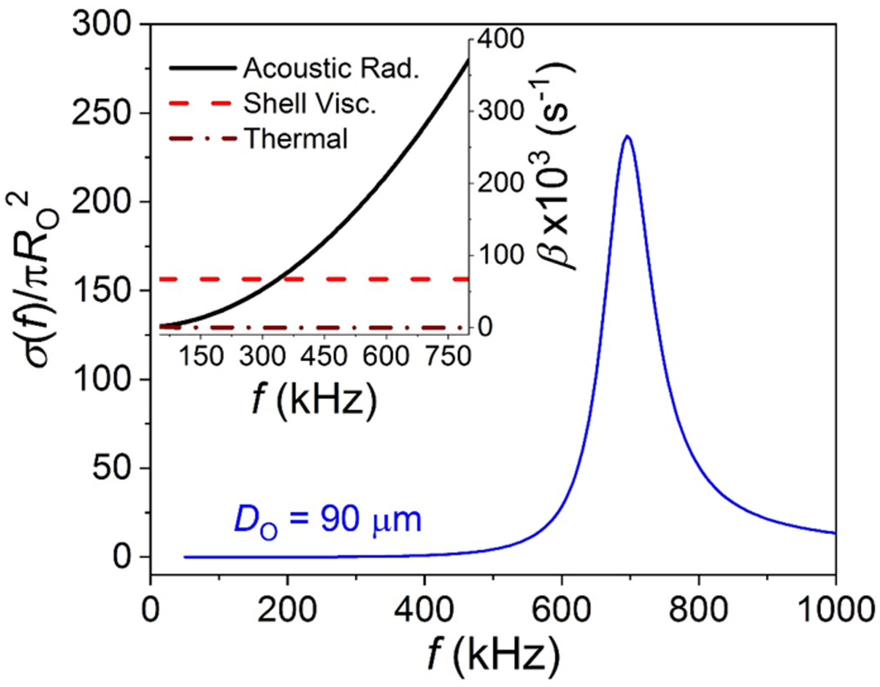


**Fig. S2** Predicted EMB scattering cross section $\sigma(f)$ versus frequency $f$ for an equilibrium diameter $D_O$ = 90 μm, which is normalized to the equilibrium cross section $\pi R_O^2$ where $R_O$ is the EMB equilibrium radius. The prediction accounts for acoustic radiation damping. On resonance, the effective scattering cross section is several orders of magnitude larger than the EMB equilibrium geometrical cross section. Inset: damping coefficient $\beta$ as a function of $f$ for damping from acoustic radiation, EMB shell viscosity, and thermal effects; damping is primarily due to acoustic radiation within the frequency range of interest (400-800 kHz).

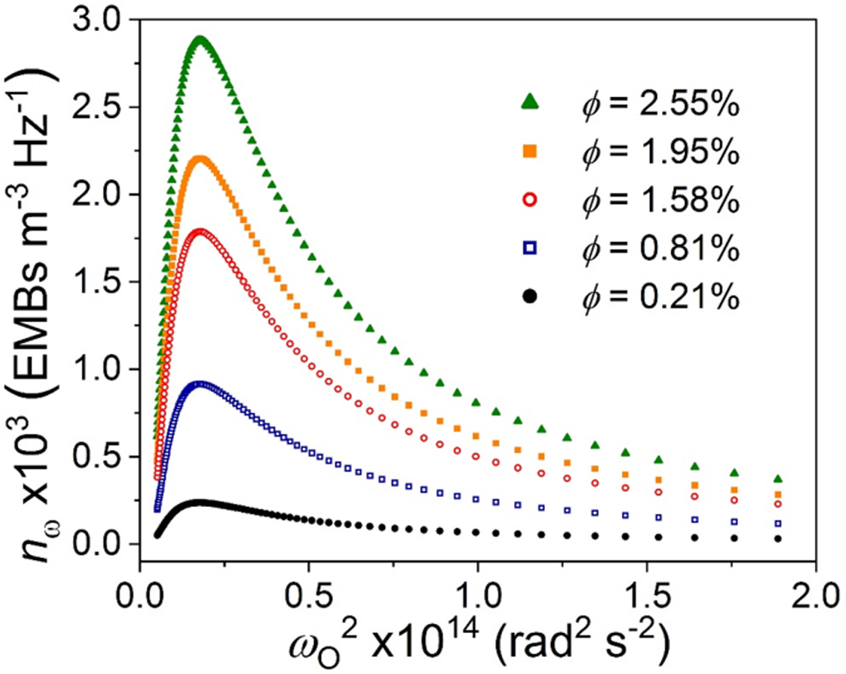


**Fig. S3** Density of states $n_\omega$ versus the square of the resonance angular frequency $\omega_O$ for five different EMB equilibrium volume fractions $\phi$ for which data is presented within the main text. Note, the $n_\omega$ versus $\omega_O{}^2$ distribution is non-Gaussian while the EMB size distribution shown in the main text's Fig. 1c is Gaussian.

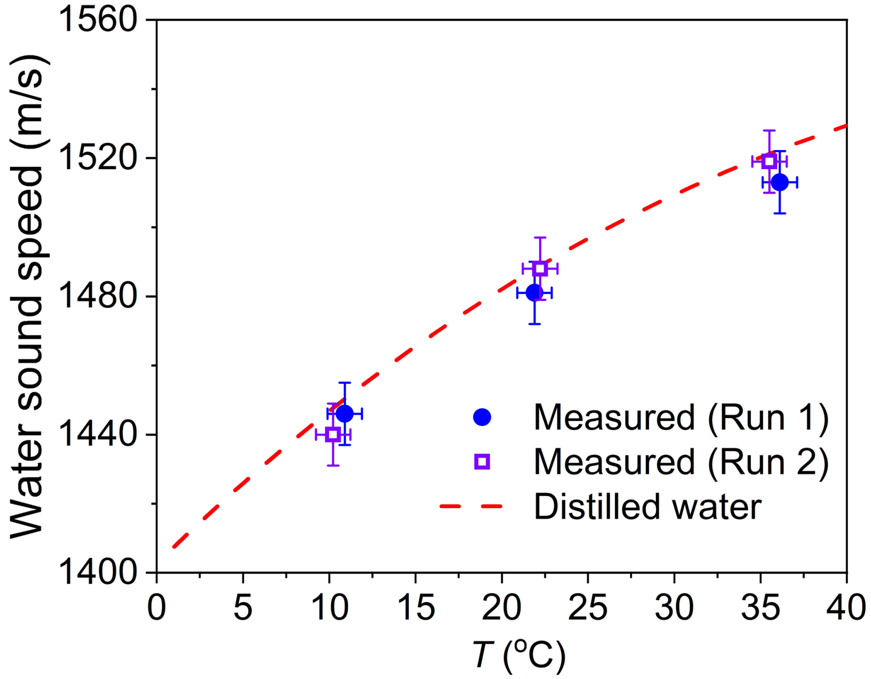


**Fig. S4** Water sound speed versus temperature *T* measured for two separate runs (solid blue circles and open violet squares, respectively) using the experimental setup shown in the main text's Fig. 1f. The sound speed is determined through a time-of-flight measurement for a 1 microsecond-wide impulse, and confirmed with a wavepacket similar to the one shown in the main text's Fig. 2a. The dashed red line is the standard published temperature-dependent sound speed profile for distilled water (see Supplementary Reference S7).

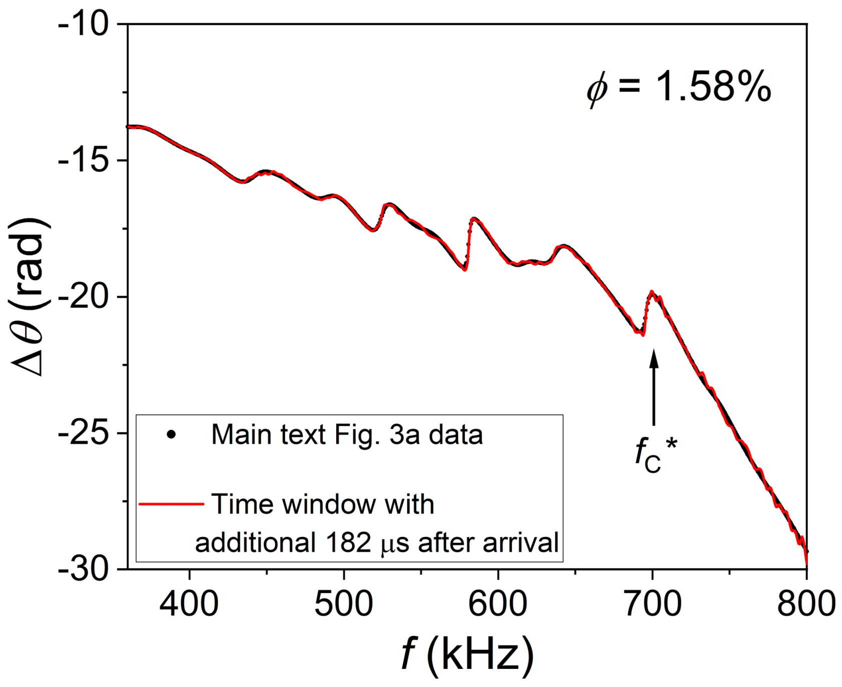


**Fig. S5** Measured change in phase angle $\Delta\theta$ versus frequency $f$ for an EMB equilibrium volume fraction $\phi$ = 1.58%. The solid black circle data set is the same data set shown in Fig. 3a of the main text. The solid red line data set corresponds to the same data, but with an additional 182 μs of time added onto the time-windowed data following the wavepacket arrival. The agreement between the two data sets indicates the results are not a result of insufficient data or signal processing.

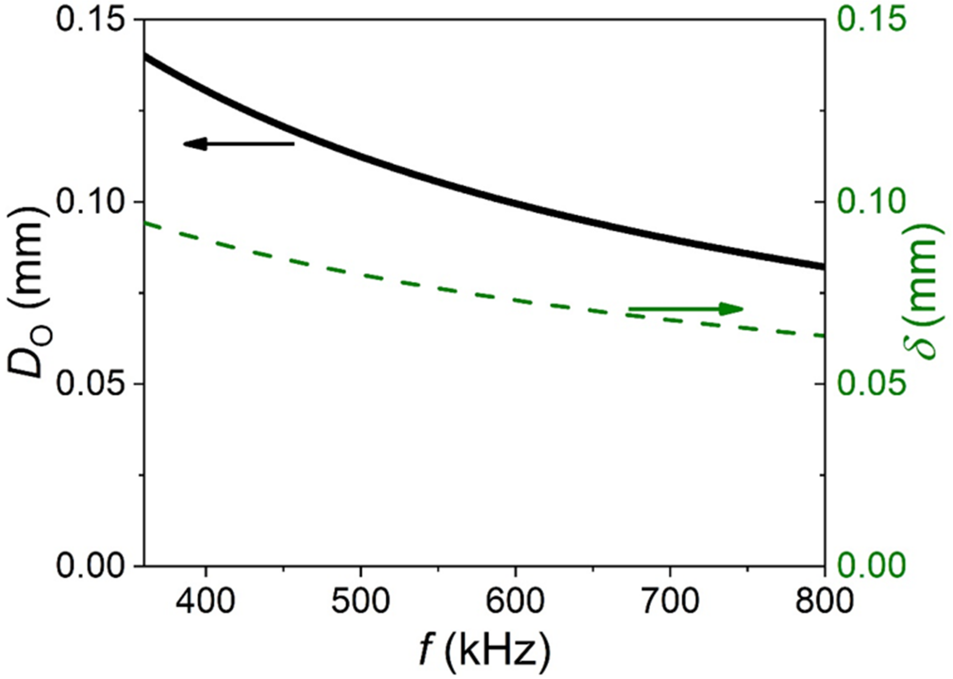


**Fig. S6** Frequency spectra for the EMB equilibrium diameter $D_O$ (solid black circles, left vertical axis) and viscous penetration depth $\delta$ (dashed green line, right vertical axis).

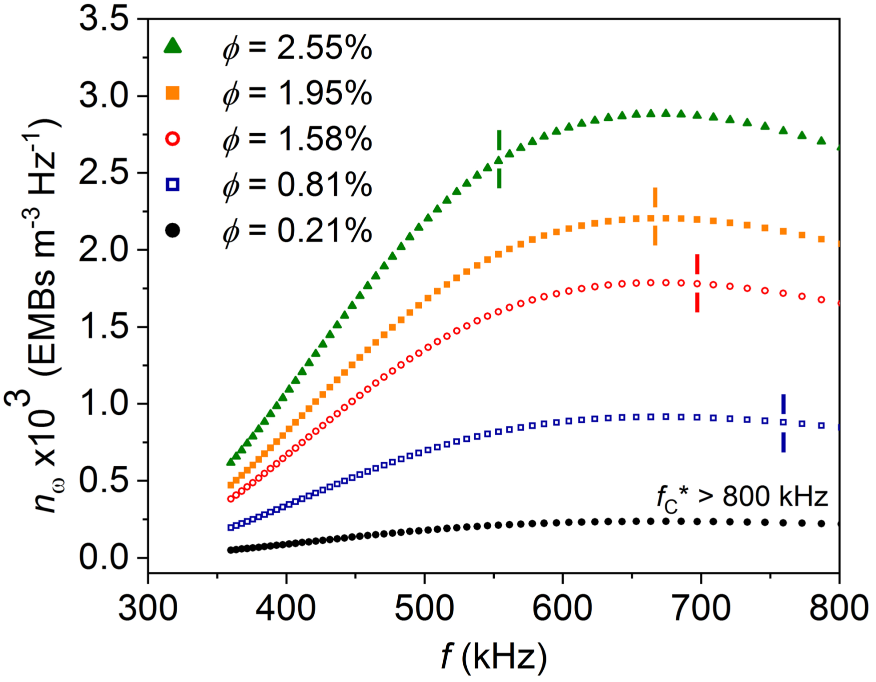


**Fig. S7** Density of states $n_\omega$ versus excitation frequency $f$ for five different EMB equilibrium volume fractions $\phi$ for which data is presented within the main text. For each data set, the vertical dashed line indicates the critical frequency $f_C$* at which a transition is observed between fluid-like and gaseous-like behavior as described within the main text.

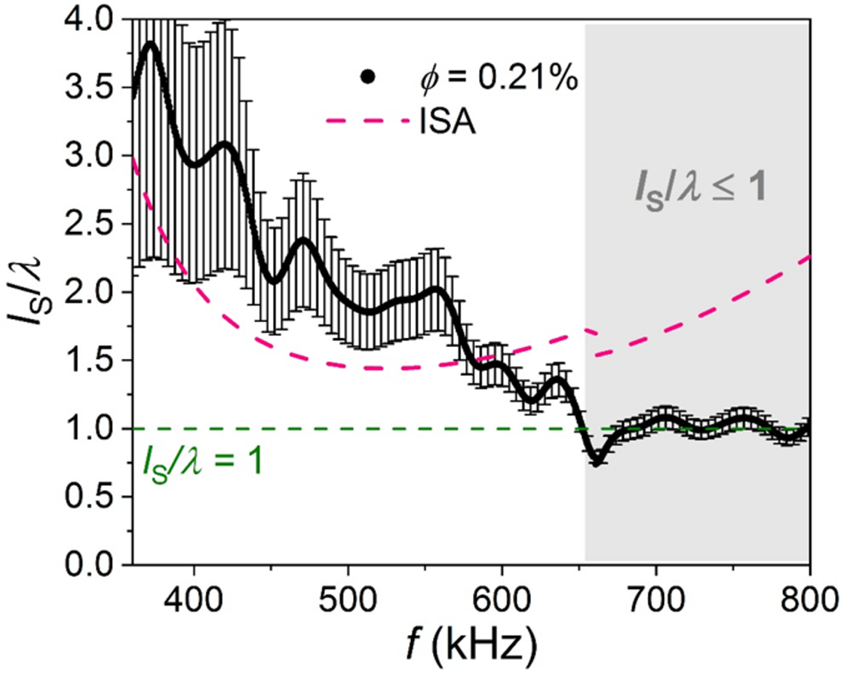


**Fig. S8** Frequency spectrum, determined from the transmitted coherent wave, for the ratio of the scattering mean free path $l_S$ to effective wavelength $\lambda$ for the sample with an EMB equilibrium volume fraction $\phi = 0.21\%$. The horizontal axis is plotted over the EMB resonance frequency range targeted by the experiments. For clarity, error bars (determined from uncertainties in the measured sound level and doped gel thickness) are shown for every 5th data point. The dashed green line is a reference to $l_S/\lambda = 1$. The shaded gray region highlights the frequency range over which we measure $l_S/\lambda \leq 1$. The dashed pink line is an independent scattering approximation (ISA) prediction based upon Eq. S20.

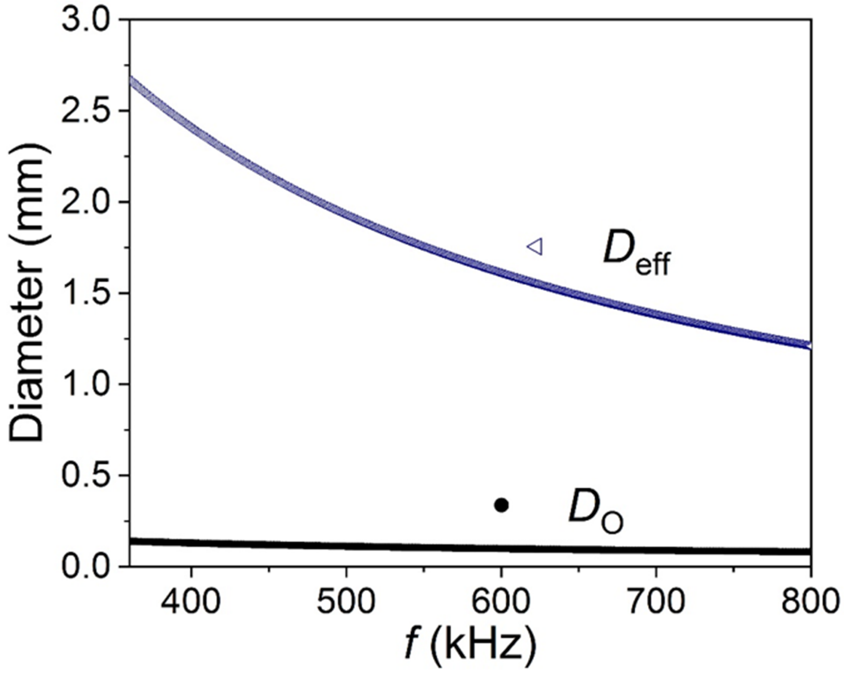


**Fig. S9** Predicted EMB on-resonance effective diameter $D_{eff}$ (open blue triangles) for a single, isolated EMB and the corresponding equilibrium diameter $D_O$ (solid black squares) versus frequency $f$.

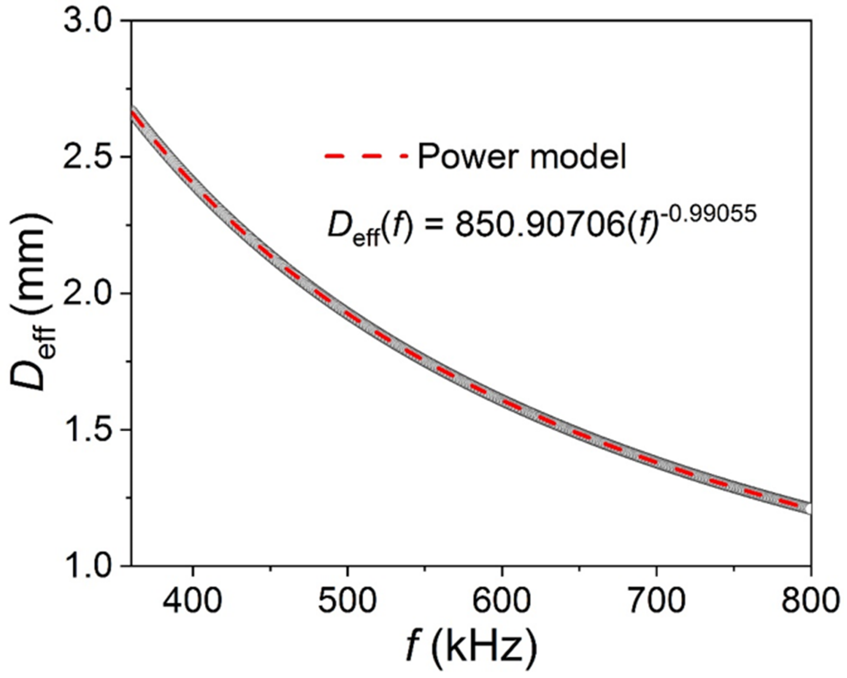


**Fig. S10** Predicted EMB on-resonance effective diameter $D_{eff}$ (open black circles) versus frequency $f$ for a single, isolated EMB along with a fit to a power model, which highlights the inverse relationship between $D_{eff}$ and $f$.

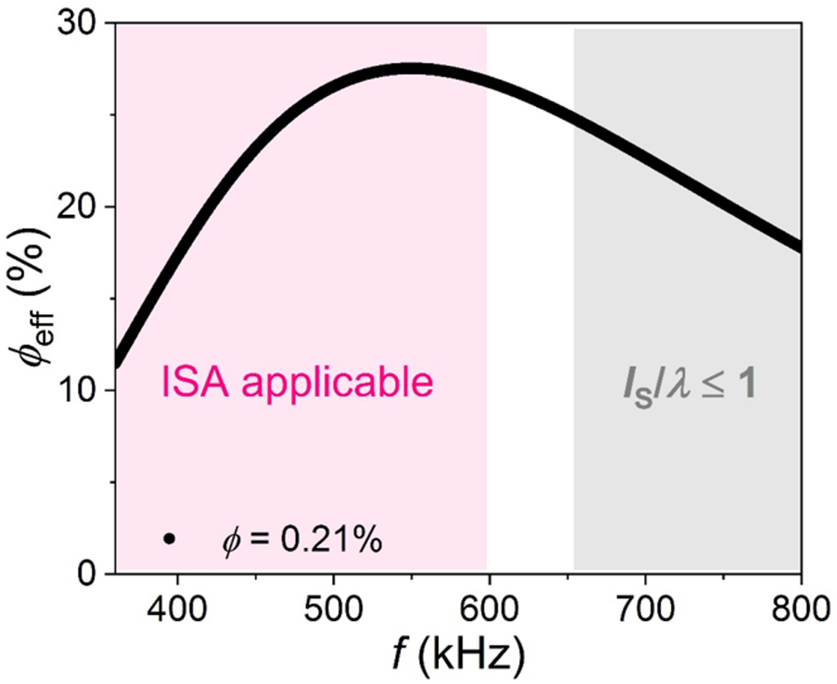


**Fig. S11** Predicted EMB effective volume fraction $\phi_{eff}$ versus frequency $f$ for an EMB equilibrium volume fraction $\phi = 0.21\%$ (solid black circles). The shaded pink region highlights the range of $f$ over which the independent scattering approximation (ISA) is qualitatively valid, which is determined from the fit shown in Supplementary Fig. S8. The shaded gray region highlights the frequency range where we measure $l_S/\lambda \le 1$.

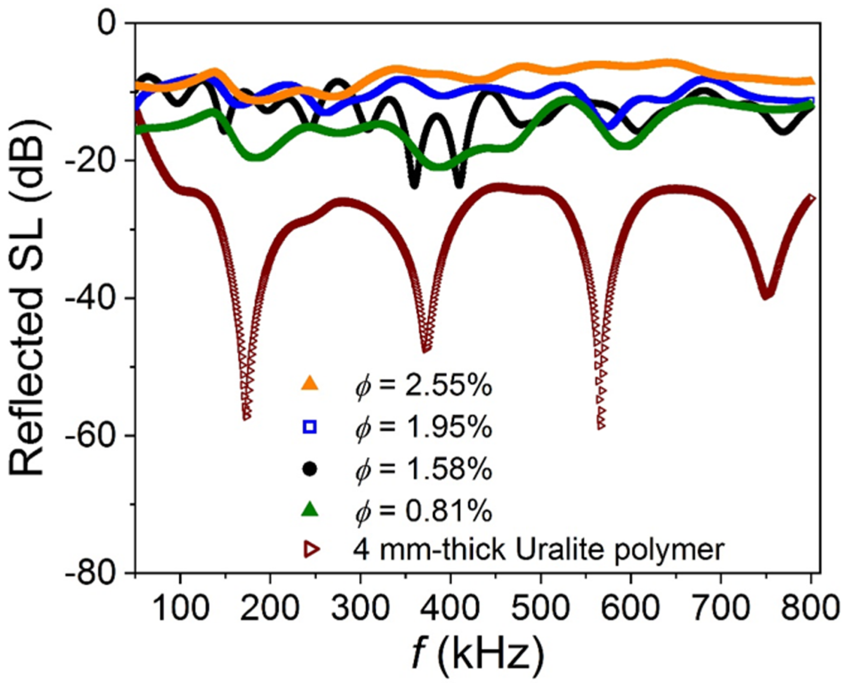


**Fig. S12** Reflected sound level (SL) versus frequency *f* for an undoped 4 mm-thick Uralite sample (open wine triangles) and for four samples with EMB equilibrium volume fractions $\phi$ = 0.81% (solid green triangles), $\phi$ = 1.58% (solid black circles), $\phi$ = 1.95% (open blue squares), and $\phi$ = 2.55% (solid orange triangles). Here, the Uralite polymer thickness is the same thickness as the undoped Uralite pocket into which the doped gel is poured for the in-water measurements.

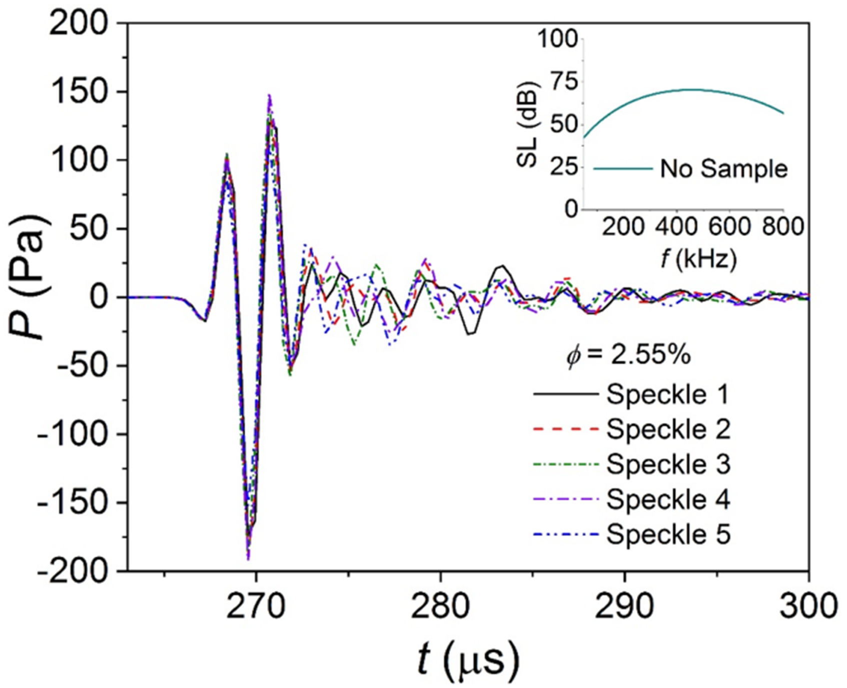


**Fig. S13** Impulse wavepacket for the late-time incoherent wave analysis (i.e., for observing diffusive and localization effects). Pressure $P$ versus time $t$ for the sample with EMB equilibrium volume fraction $\phi = 2.55\%$ recorded during a transmission measurement and for different speckles. The incident wavepacket is based on a Gaussian first derivative, which provides a narrow impulse. The coherent wave is observable for $266\ \mu s < t < 272\ \mu s$, and is followed by the incoherent field. The incoherent field data shown here was used to obtain the data shown in Supplementary Fig. 14(e) and Supplementary Fig. S15(b) for $\phi = 2.55\%$, and similar data sets were collected for the $\phi = 1.58\%$ and 1.95% samples to obtain the data shown in Supplementary Fig. S14(a)-(d) and for the $\phi = 0.21\%$ sample to obtain the data shown in Supplementary Fig. S15(a). Inset: sound level SL frequency spectrum for the Gaussian first derivative wavepacket obtained during the water reference (no sample) measurement.

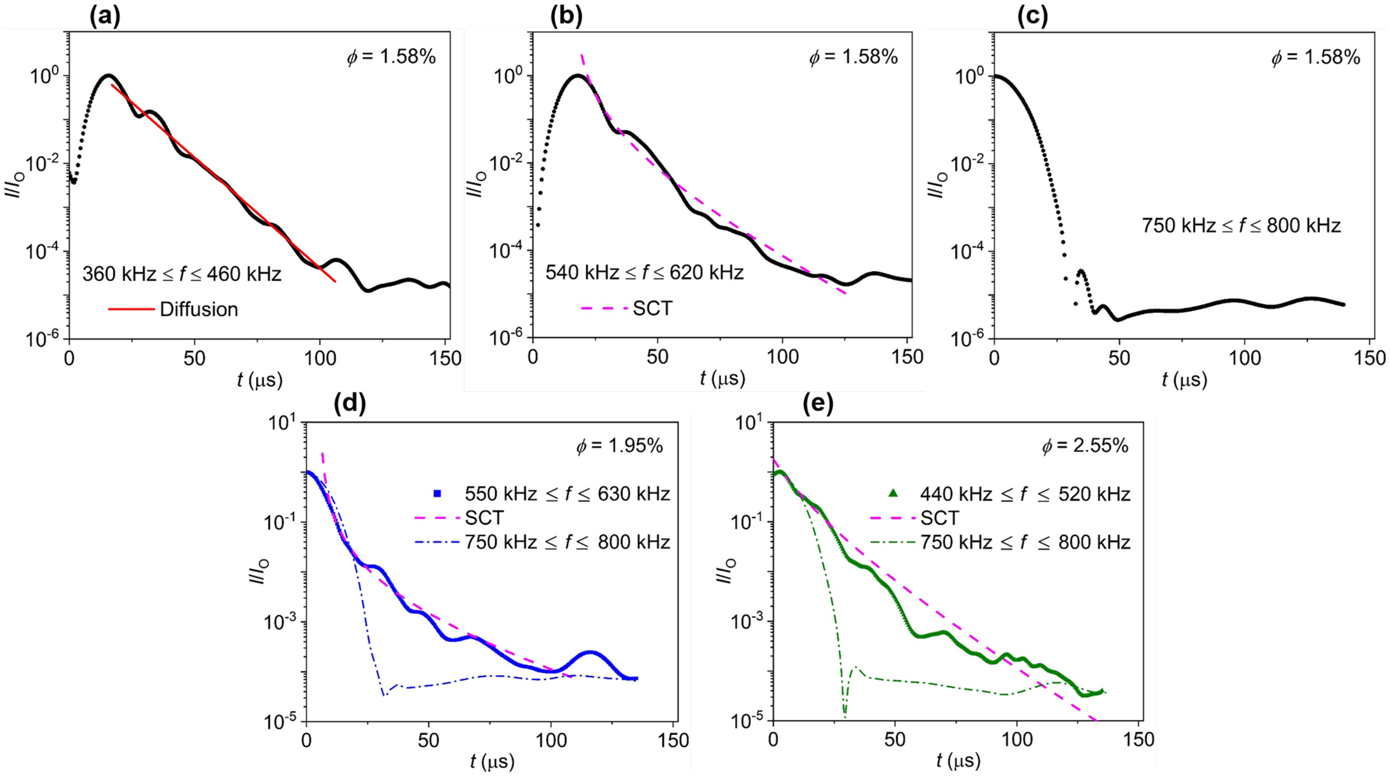


**Fig. S14** Incoherent wave analysis across the two asymptotic regimes: fluid-like (including diffusive states) and quasi-gaseous ($f > f_C$*; in (c)-(e) the 750-800 kHz frequency range corresponds to the quasi-gaseous phase). The normalized transmitted intensity peak envelope $I/I_O$ (on a semi-logarithmic plot) is plotted versus time $t$ for the incoherent energy and for the three doped samples for which data is presented in Fig. 2 of the main text: $\phi = 1.58\%$ in (a)-(c), $\phi = 1.95\%$ in (d), and $\phi = 2.55\%$ in (e). Incoherent wave data is digitally filtered to target those frequency ranges shown in each figure part based upon the $l_S/\lambda$ data shown in Fig. 2(d)-(f) of the main text. $I/I_O$ is found from averaging over 11 different speckle measurements. Normalization is done so the input pulse peak is unity, and then so that in each figure part the maximum occurs at $I/I_O = 1$. The time ranges are shifted from the experiment time so the maximum in $I/I_O$ occurs shortly after $t = 0$ s. The solid red line in (a) is a linear fit (diffusion) with the characteristic diffusion time $\tau_D$ serving as a free fitting parameter. Dashed magenta lines in (b), (d), and (e) are fits to the localization self-consistent theory (SCT) with $D_B$, $\xi$, and $\tau_a$ (bare diffusion coefficient, localization length, and characteristic absorption time, respectively) serving as free fitting parameters.

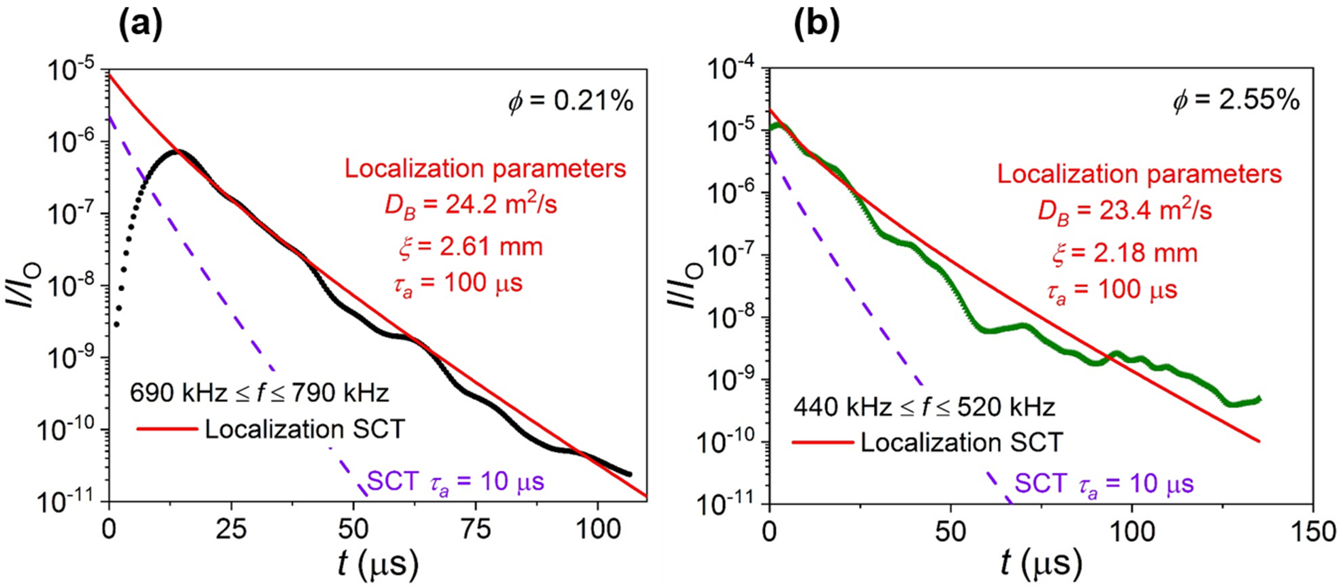


**Fig. S15** Wave localization effects (and SCT analysis) in EMB-doped gel for when $l_S/\lambda < 1$. Normalized transmitted intensity peak envelope $I/I_O$ (on a semi-logarithmic plot) plotted versus time $t$ for the incoherent energy and for the two doped samples that represent the full range of $\phi$ over which the behavior of $f_C$* versus $\phi$ is studied within the main text (Fig. 5). Incoherent wave data is digitally filtered to target those frequency ranges shown in each figure part where $l_S/\lambda < 1$. $I/I_O$ is found from averaging over 11 different speckle measurements. Normalization is done so the input pulse peak is unity. Data shown in (b) is the same data set shown in Supplementary Fig. 14(e). The time ranges are shifted from the experiment time so the maximum in $I/I_O$ occurs shortly after $t = 0$ s. Solid red lines are fits to the self-consistent theory (SCT) of localization with $D_B$, $\xi$, and $\tau_a$ (bare diffusion coefficient, localization length, and characteristic absorption time, respectively) serving as free fitting parameters, and the resultant values from the SCT fits are shown in red. Dashed purple lines correspond to setting $\tau_a$ = 10 μs in the SCT fit while keeping all other parameters fixed at the values specified.

**Supplementary References:**

S1. Chen, J., Hunter, K. S., & Shandas, R. Wave scattering from encapsulated microbubbles subject to high-frequency ultrasound: contribution of higher-order scattering modes. *J. Acoust. Soc. Am.* **126,** 1766-1775 (2009).

S2. Matis, B. R. *et al*. Observation of a transition to a localized ultrasonic phase in soft matter. *Commun. Phys.* **5,** 21 (2022).

S3. Matis, B. R. *et al*. Critical role of a nanometer-scale microballoon shell on bulk acoustic properties of doped soft matter. *Langmuir* **36,** 5787-5792 (2020).

S4. Kinsler, L. E., Frey, A. R., Coppens, A. B. & Sanders, J. V. *Fundamentals of Acoustics* (John Wiley & Sons, Inc., 1982), p. 228.

S5. Tan, L., Pan, J. & Wan, A. Shear and extensional rheology of polyacrylonitrile solution: effect of ultrahigh molecular wight polyacrylonitrile. *Colloid Polym. Sci*. **290,** 289-295 (2012).

S6. Khismatullin, D. B. & Nadim, A. Radial oscillations of encapsulated microbubbles in viscoelastic liquids. *Phys. Fluids* **14,** 3534-3557 (2002).

S7. Kinsler, L. E., Frey, A. R., Coppens, A. B. & Sanders, J. V. *Fundamentals of Acoustics* (John Wiley & Sons, Inc., 1982), p. 107.

S8. Biot, M. A. Theory of propagation of elastic waves in a fluid-saturated porous solid. I. low-frequency range. *J. Acoust. Soc. Am*. **28,** 168-178 (1956).

S9. Biot, M. A. Theory of propagation of elastic waves in a fluid-saturated porous solid. II. Higher frequency range. *J. Acoust. Soc. Am*. **28,** 179-191 (1956).

S10. Tallon, B., Brunet, T. & Page, J. H. Impact of strong scattering resonances on ballistic and diffusive wave transport. *Phys. Rev. Lett*. **119,** 164301 (2017).

S11. Page, J. H., Schriemer, H. P., Bailey, A. E. & Weitz, D. A. Experimental test of the diffusion approximation for multiply scattered sound. *Phys. Rev. E* **52,** 3106-3114 (1995).

S12. Korneev, V. A. & Johnson, L. R. Scattering of P and S waves by a spherically symmetric inclusion. *Pure and Appl. Geophys*. **147,** 675-718 (1996).

S13. Rimberg, A. J. & Westervelt, R. M. Temporal fluctuations of multiply scattered light in a random medium. *Phys. Rev. B* **38,** 5073(R) (1988).

S14. Stephen, M. J. Temporal fluctuations in wave propagation in random media. *Phys. Rev. B* **37,** 1-5 (1988).

S15. Mirlin, A. D. Statistics of energy levels and eigenfunctions in disordered systems. *Phys. Rep*. **326,** 259-382 (2000).

S16. Skipetrov, S. E. & van Tiggelen, B. A. Dynamics of Anderson localization in open 3D media. *Phys. Rev. Lett*. **96,** 043902 (2006).

S17. Hu, H., Strybulevych, A., Page, J. H., Skipetrov, S. E. & van Tiggelen, B. A. Localization of ultrasound in a three-dimensional elastic network. *Nat. Phys*. **4,** 945-948 (2008).
